# Supplementary material for: Applicability and cost-effectiveness of the Systolic Blood Pressure Intervention Trial (SPRINT) in the Chinese population: A cost-effectiveness modeling study
Source: PLoS Med. 2021 Mar 4;18(3):e1003515. doi: 10.1371/journal.pmed.1003515 (PMC7971845; doi:10.1371/journal.pmed.1003515)
Supplement: S1 CHEERS Checklist — (DOCX) [file pmed.1003515.s001.docx]

**S1** CHEERS checklist

| Section/Item |  | Recommendation | Section /Paragraph |
| --- | --- | --- | --- |
| **Title and abstract** | | | |
| Title | 1 | Identify the study as an economic evaluation or use more specific terms such as cost-effectiveness analysis`, and describe the interventions compared. | Title |
| Abstract | 2 | Provide a structured summary of objectives, perspective, setting, methods (including study design and inputs), results (including base case and uncertainty analyses) and conclusions. | Abstract |
| **Introduction** | | | |
| Background and objectives | 3 | Provide an explicit statement of the broader context for the study. | Introduction/Para 1-2 |
|  |  | Present the study question and its relevance for health policy or practice decision. | Introduction/Para 3 |
| **Methods** | | | |
| Target Population And  Subgroups Populations | 4 | Describe characteristics of the base case population and subgroups analyzed including why they were chosen. | Data source & study population/Para 1/S3 Table |
| Setting and Location | 5 | State relevant aspects of the system(s) in which the decision(s) need(s) to be made. | Introduction/Para 3 |
| Study Perspective | 6 | Describe the perspective of the study and relate this to the costs being evaluated. | Cost and utility weights / Para 1 |
| Comparator | 7 | Describe the interventions or strategies being compared and state why they were chosen. | Introduction/Para 3 |
| Time Horizon | 8 | State the time horizon(s) over which costs and consequences are being evaluated and say why appropriate. | Introduction/Para 3 |
| Discount Rate | 9 | Report the choice of discount rate(s) used for costs and outcomes and say why appropriate. | Cost and utility weights / Para 1 |
| Choice of Health Outcomes | 10 | Describe what outcomes were used as the measure(s) of benefit in the evaluation and their relevance for the type of analysis performed. | Design of the health economic model/ Para 1  Cost and utility weights / Para 1-2 |
| Measurement of Effectiveness | 11a | Single Study-Based Estimates: Describe fully the design features of the single effectiveness study and why the single study was a sufficient source of clinical effectiveness data. | NA |
|  | 11b | Synthesis-based Estimates: Describe fully the methods used for identification of included studies and synthesis of clinical effectiveness data. | Transition probabilities /Para 1  S4 table |
| Measurement and Valuation  Of Preference-Based Outcomes | 12 | If applicable, describe the population and methods used to elicit preferences for outcomes. | NA |
| Estimating Resources and Costs | 13a | Single Study-based Economic evaluation: Describe approaches used to estimate resource use associated with the alternative interventions. Describe primary or secondary research method for valuing each resource item in terms of its unit cost. Describe any adjustments made to approximate to opportunity costs. | NA |
|  | 13b | Model-based Economic Evaluation: Describe approaches and data sources used to estimate resource  use associated with model health states. Describe primary or secondary research methods for valuing  each resource items in terms of its unit cost. Describe any adjustments made to approximate to  opportunity costs. | S1 appendix text- Cost /Para 1-2  S5 table |
| Currency, Price Date and  Conversion | 14 | Report the dates of the estimated resource quantities and unit costs. Describe methods for adjusting  estimated unit costs to the year of reported costs if necessary. Describe methods for converting costs  into a common currency base and the exchange rate. | Cost and utility weights / Para 1  S1 appendix text- Cost /Para 1-2 |
| Choice of model | 15 | Describe and give reasons for the specific type of decision-analytic model used. Providing a figure to show model structure is strongly recommended. | Model structure /Para 1  S1 Fig |
| Assumption | 16 | Describe all structural or other assumptions underpinning the decision-analytic model. | Cost and utility weights / Para 1  S4 table S5 table  Model structure /Para 1 |
| Analytic Methods | 17 | Describe all analytic methods supporting the evaluation. This could include methods for dealing with skewed, missing, or censored data, Extrapolation methods, methods for pooling data, approaches to validate or make adjustments (e.g., half-cycle corrections) to a model and method for handling population heterogeneity and uncertainty. | Base case and sensitivity Analyses/para 1-2  S1 appendix text- model structure /Para 1 |
| **Results** | | | |
| Study parameters | 18 | Report the values, ranges, References and if used, probability distributions for all parameters. Report reasons or sources for distributions used to represent. Uncertainty where appropriate. Providing a table to show the input values is strongly recommended. | S4 table S5 table |
| Incremental costs and  outcomes | 9 | For each intervention, report mean values for the main categories of estimated costs and outcomes of interest, as well as mean differences between the comparator groups. If applicable, report incremental cost-effectiveness ratios. | Table 1/S8 Table |
| Characterizing  uncertainty | 20a | Single study-based economic evaluation: Describe the effects of sampling uncertainty for estimated incremental cost, incremental effectiveness, and incremental cost effectiveness, together with the impact of methodological assumptions (e.g. discount rate, study perspective). | NA |
|  | 20b | Model-based economic evaluation: Describe the effects on the results of uncertainty for all input parameters, and uncertainty related to the structure of the model and assumptions. | S3 Fig, S4 Fig, S5 Fig |
| Characterizing  Heterogeneity | 21 | If applicable, report differences in costs, outcomes or cost- effectiveness that can be explained by  variations between subgroups of patients with different baseline characteristics or other observed  variability in effects that are not reducible by more information. | Table 1, S3 Fig |
| **Discussion** | | | |
| Study Findings, Limitations,  Generalizability and Current  Knowledge | 22 | Summarize key study findings and describe how they support the conclusions reached. Discuss limitations and the generalizability of the findings and how the findings fit with current knowledge. | Discussion/Para 1and Para 4 |
| **Other** | | | |
| Source of Funding | 23 | Describe how the study was funded and the role of the funder in the identification, design\conduct and reporting of the analysis. Describe other non-monetary sources of support. | Financial disclosure |
| Conflicts of Interest | 24 | Describe any potential for conflict of interest among study contributor’s in accordance with journal policy. In the absence of a journal policy, we recommend authors comply with International Committee of Medical Journal Editors’ recommendations | Competing Interests |

CHEERS checklist - Items to include when reporting economic evaluations of health interventions
