# Supplementary figures and images for: Applicability and cost-effectiveness of the Systolic Blood Pressure Intervention Trial (SPRINT) in the Chinese population: A cost-effectiveness modeling study
Source: PLoS Med. 2021 Mar 4;18(3):e1003515. doi: 10.1371/journal.pmed.1003515 (PMC7971845; doi:10.1371/journal.pmed.1003515)

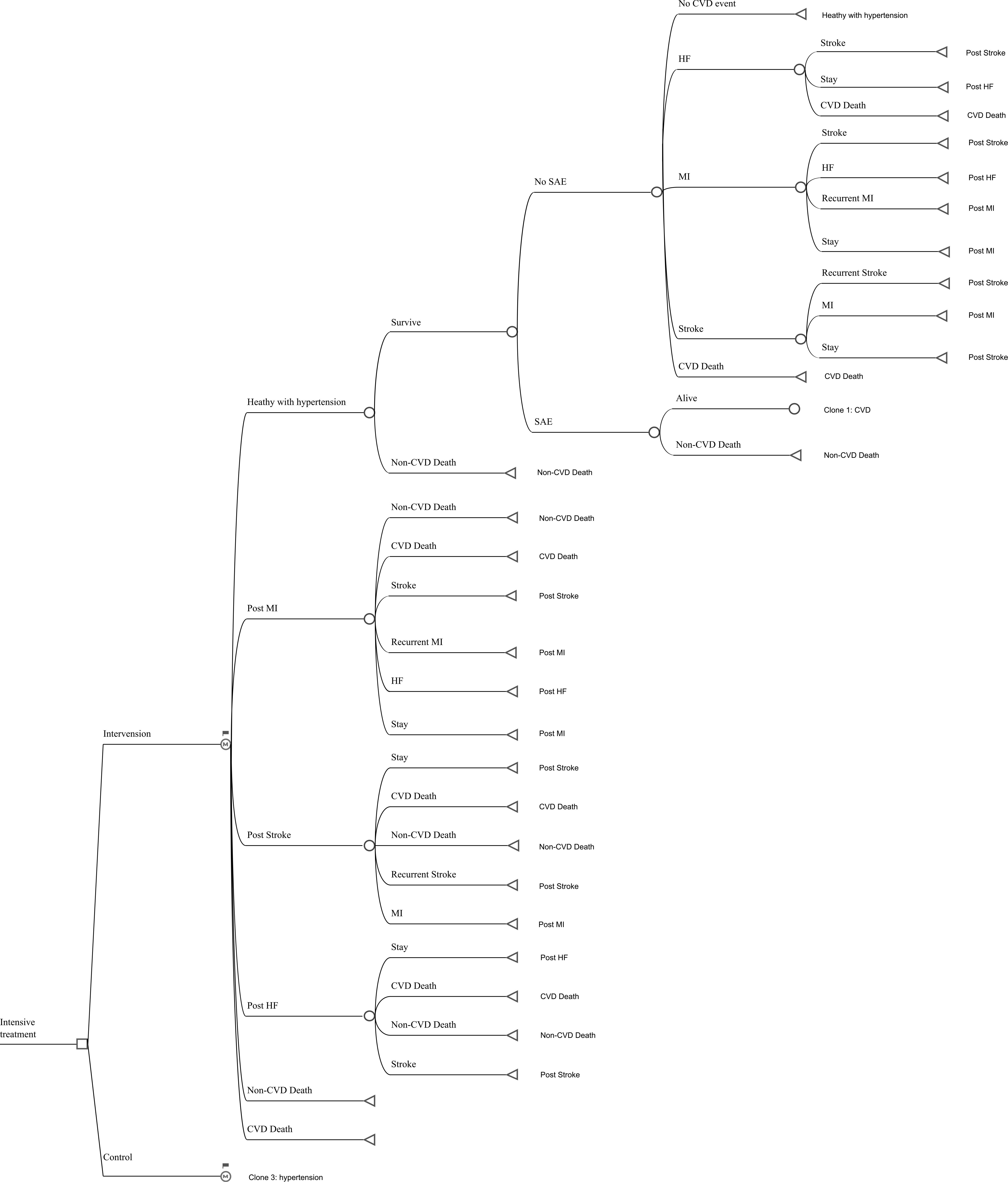

Supplement: S1 Fig — HF, heart failure; MI, myocardial infarction. (TIF) [file pmed.1003515.s002.tif]

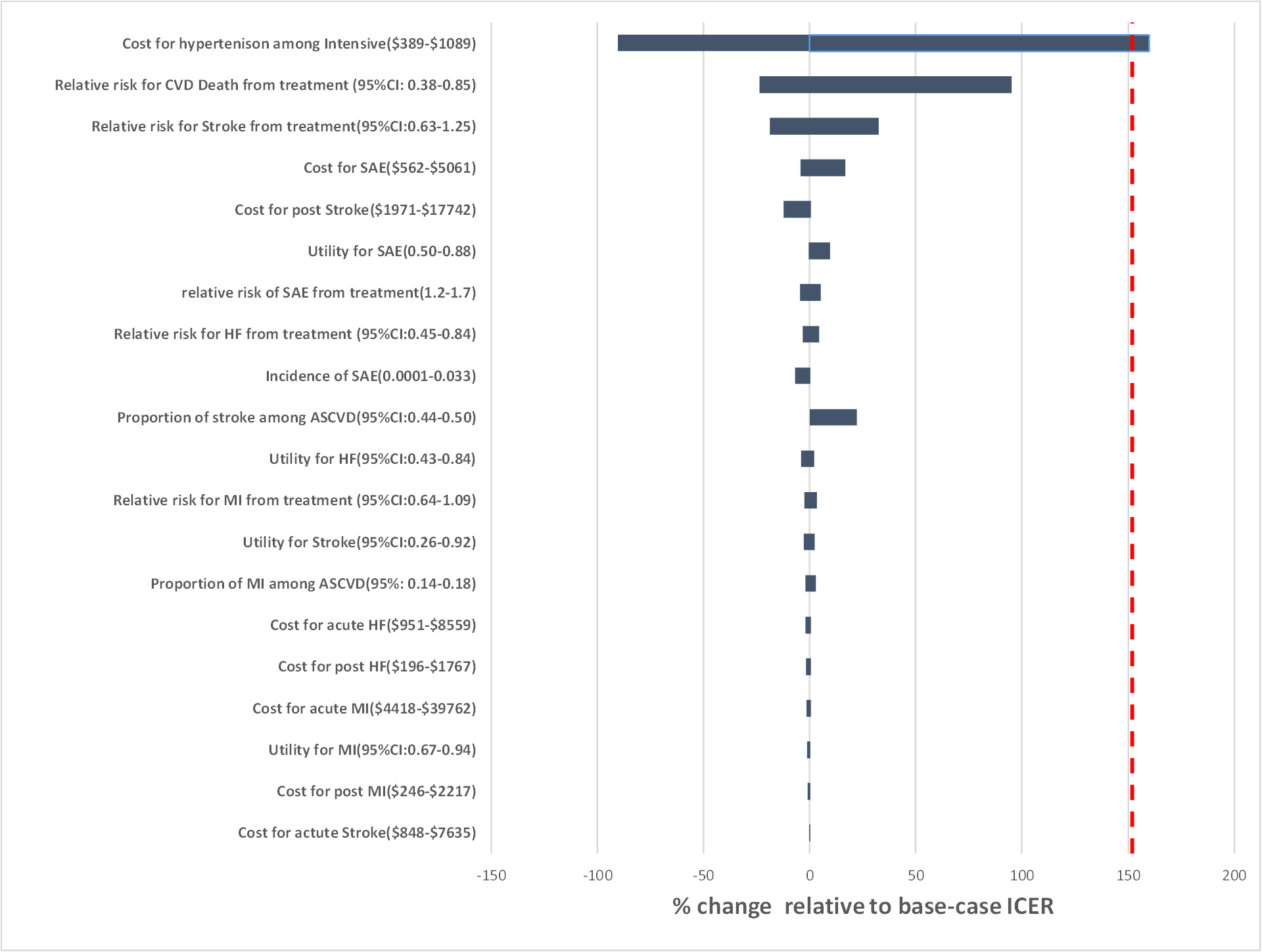

Supplement: S2 Fig — Red dashed line: 1 GDP per capita. (TIF) [file pmed.1003515.s003.tif]

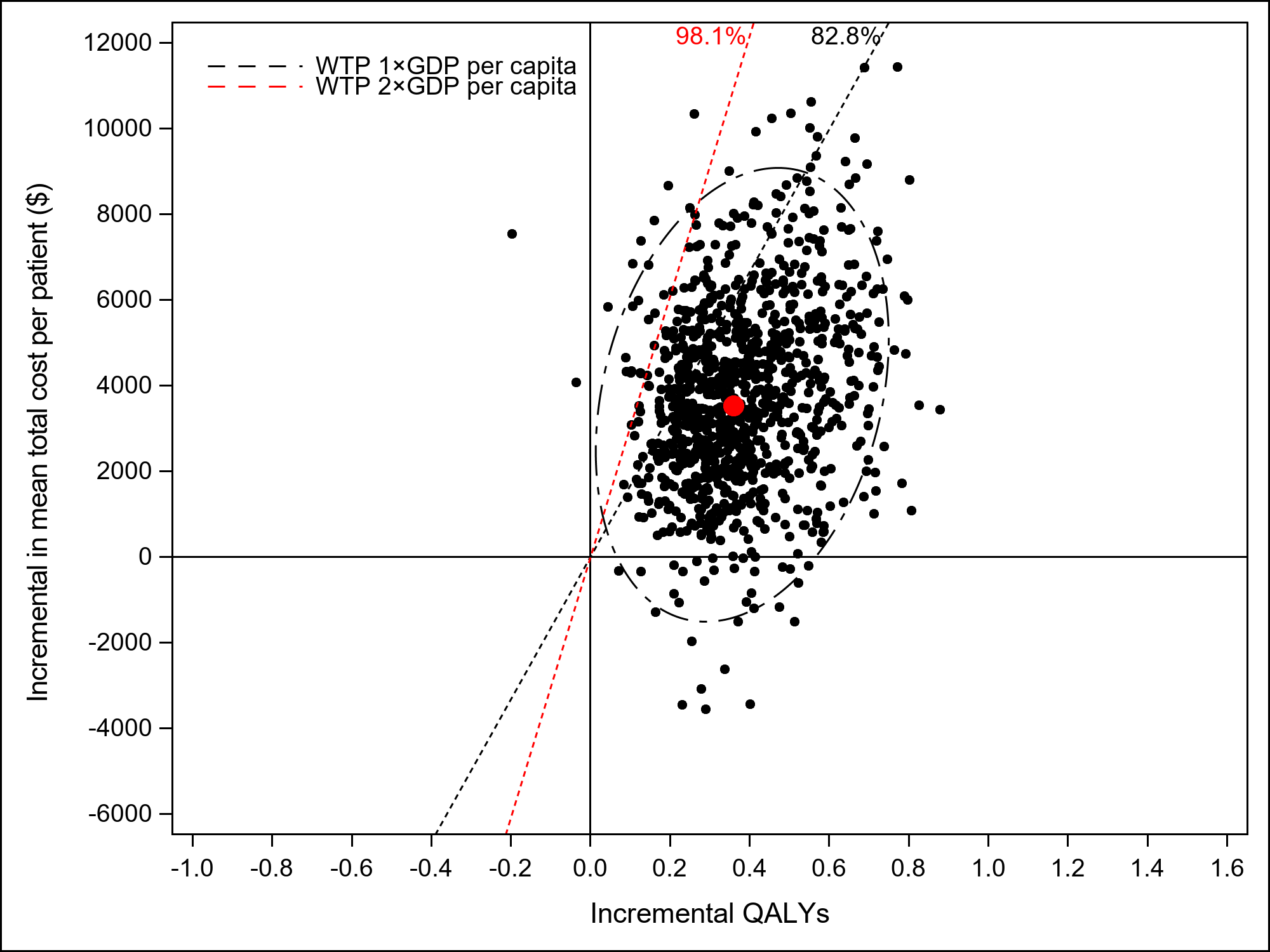

Supplement: S3 Fig — (TIFF) [file pmed.1003515.s004.tiff]

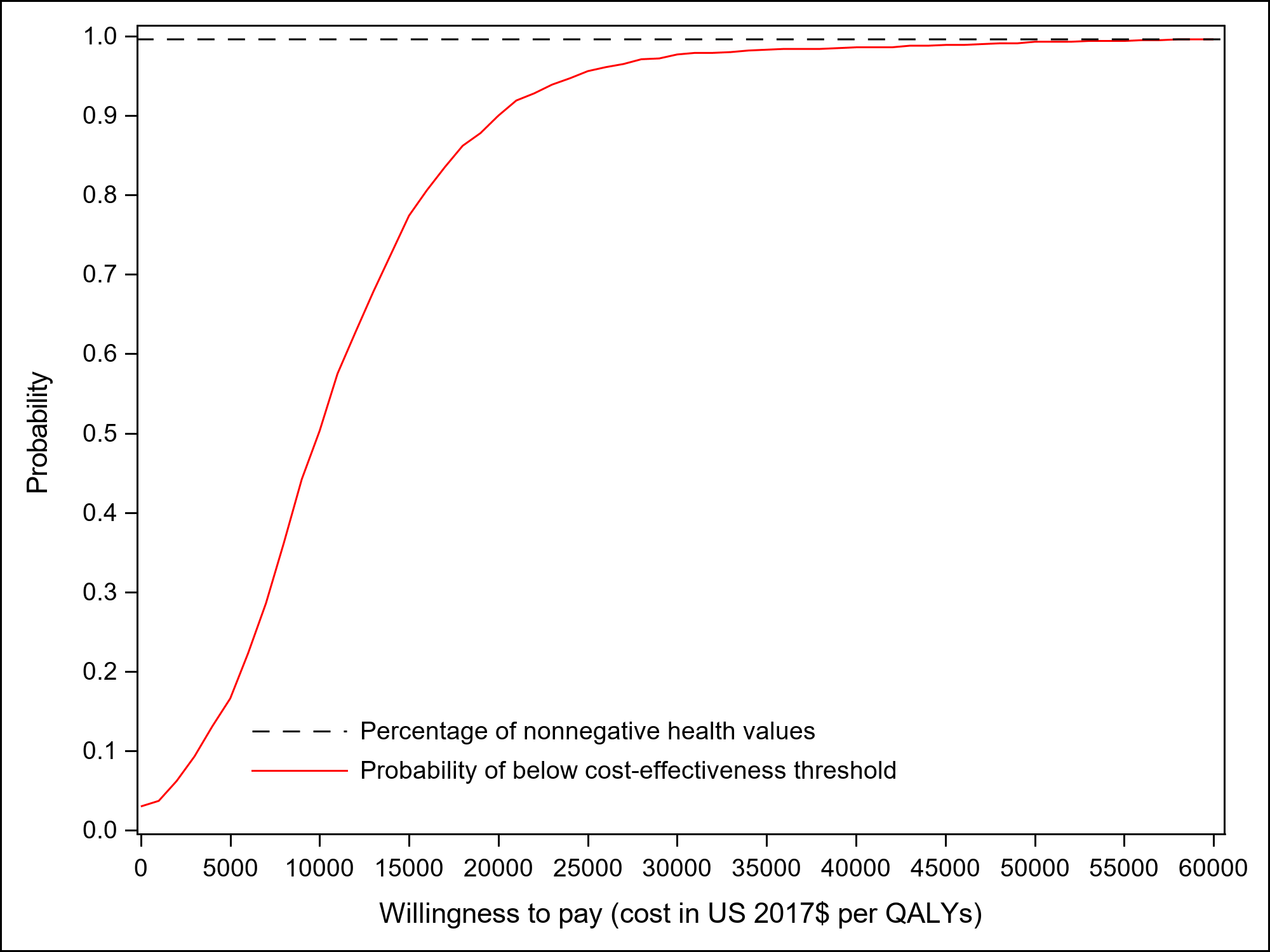

Supplement: S4 Fig — (TIFF) [file pmed.1003515.s005.tiff]
