# Supplementary material for: Applicability and cost-effectiveness of the Systolic Blood Pressure Intervention Trial (SPRINT) in the Chinese population: A cost-effectiveness modeling study
Source: PLoS Med. 2021 Mar 4;18(3):e1003515. doi: 10.1371/journal.pmed.1003515 (PMC7971845; doi:10.1371/journal.pmed.1003515)
Supplement: S1 Text — (DOCX) [file pmed.1003515.s006.docx]

**Supporting information**

**Table A** Characteristics among the overall and those meeting SPRINT eligibility ^a^

**Table B** Numbers of overall participants and participants meeting each sequential SPRINT eligibility criterion, millions

**Table C** Probability of new events after disease and treatment effect from intensive BP treatment

**Table D** Annual event cost and Quality of life weight

**Table E** Comparison of the 5-year incidences or life-years with published data

**Table F** 5 year and life-time events difference for different outcomes from base-case analysis

**Table G** Total costs of intensive BP control and standard BP treatment

**Study variables and definition**

The data used in the present study are from three sources: 1) a questionnaire administered through face-to-face interviews; 2) a physical examination carried out by trained interviewers; 3) laboratory blood tests.

BP was taken by an attended automated BP device (Omron model HEM-7200). We calculated the average SBP and DBP based on 3 blood pressure measurements (approximately 45s apart) for each person. Additionally, we obtained information on demographic characteristics (i.e., age, educational level[None, Primary, Secondary/High School, College/University or above], Sex); self-reported data on medical history by the question of ‘Have you been diagnosed with hypertension/diabetes/stroke/heart failure/CHD by a doctor?’; Smoking status; BMI [Overweight, defined as 25≤BMI<30 Kg/m^2^; Obese, defined as BMI≥30 Kg/m^2^]; laboratory tests for serum creatinine, total cholesterol, high density lipoprotein cholesterol). Estimated glomerular filtration rate (eGFR) was calculated using the *Modification of Diet in Renal Disease* equation. The Framingham risk score was calculated using the *Framingham cardiovascular disease event equation*. Diabetes was defined as haemoglobin A_1C_≥6.5%, use of diabetes medication, or self-reported history of diabetes. Hypertension was defined as a SBP of 140 mmHg or more, or a DBP of 90 mmHg or more, or self-reported use of antihypertensive treatment. Detailed information about the data quality management has been estimated previously[[1](#_ENREF_1)].

**Model structure:**

Unlike our previous work using a Markov cohort model[[2](#_ENREF_2)], we developed a microsimulation model to keep track of individual trajectories based on individual-specific transition probabilities. Eligible hypertensive individuals may start the model with/without serious adverse events, but all were followed along the disease paths until they turned 100 years old or died. After each cycle, subjects were redistributed to one of the following 9 health states: (1) hypertension; (2) stroke; (3) post stroke; (4) Myocardial infarction (MI); (5) post MI; (6) heart failure (HF); (7) post HF; (8) CVD mortality and (9) non-CVD mortality. The overall structure of our simulation model can be found in **Figure A**. For each individual disease path, patients may either survive or die from a disease suffered within a period. During the first year, patients might experience more than one event (e.g. the occurrence of Stroke after MI). After an event, patients transitioned into one of the following chronic health states: post MI, post stroke and post HF. While being in these states, patients might suffer from further CVD events. Schematic depictions for each event could be found in appendix of our previous work[[2](#_ENREF_2)]*.*  Also, in our study, we assumed subjects returned to standard management once they experienced cardiovascular events for both blood pressure managements. The model cycle length for our study was set to 1 year with half-correction applied.

**Transition probability**

For individuals who experienced a first non-fatal cardiovascular event, this might be followed by a secondary event or chronic conditions. However, since there are no particular risk equations to calculate these disease risks among the Chinese population, we extracted the transition probabilities for the subsequent events (within or >1 year after a primary event), together with their distributions, from relevant disease registries and/or meta-analysis reports (see **Table C**). In terms of the rates of adverse events from antihypertensive medications, we used the rate from a meta-analysis report[[3](#_ENREF_3)], which was also adopted in two other cost-effectiveness analyses[[4](#_ENREF_4), [5](#_ENREF_5)].

Age- and sex-specific all-cause/CVD mortality from Chinese National Statistical Office in 2013 were derived as background mortality and was adjusted to account for the higher risk from hypertension in our study [25]. However, the non-CVD mortality risks, which were recalculated after exclusion of CVD death from the total mortality, have been assumed to be unaffected by the states in our model. After calculating the event rates, we converted them into transition probabilities. The values for the transition probabilities and their distributions are summarised in **Table C.**

The relationship between the event rate (r) and the transition probability ($\rho$) for time period t is given by: $\rho=1-e^{-rt}$.

**Cost:**

Annual median cost and prescription frequency for 62 oral medications by generic names was estimated in a nationwide cross-sectional survey[[6](#_ENREF_6)]. In our study, we focused on the four standard antihypertensive drug classes (i.e., thiazide diuretic, angiotensin converting enzyme inhibitor, beta blocker, and long-acting calcium channel blocker). We adopted the combinations of medications recommend in Chinese guidelines for the management of hypertension 2018[[7](#_ENREF_7)]. It is reported in SPRINT study[[8](#_ENREF_8)] that 2.7 and 1.8 antihypertensive medications were received for intensive and usual group, respectively. Therefore, the cost for the combinations of standard dose medications were taken as the cumulative cost of the individual drugs which were prescribed most frequently[[6](#_ENREF_6)].

The cost of events within the first year were calculated by adding the costs of hospitalization extracted from the China Health Statistics Yearbook 2018[[9](#_ENREF_9)] to the average annual cost of post disease. Detailed information for each component is described below: cost of hospitalization for patients with a stroke is RMB 18,524.6 for haemorrhages stroke and RMB 9,607 for ischemic stroke, respectively[[10](#_ENREF_10)], and average annual cost for post stroke was RMB: 9,009[[11](#_ENREF_11)]; For the cost of hospitalized MI, we chose the same method as the Yanfei Wu’ study[[12](#_ENREF_12)], which assumed 50% surgery (RMB 61,051 for CABG treatment) and 50% nonsurgery treatment (RMB 27,552.5 for PCI treatment). The average annual cost used for post MI was RMB 2,616[[13](#_ENREF_13)]; Hospitalized cost for patients with HF is RMB 8,014.1, and cost of post HF was estimated to be RMB1,837 [[14](#_ENREF_14)]. The cost of serious adverse events we estimate as RMB 5,971 after inflating to 2017[[5](#_ENREF_5)]. Regarding the range of the costs, as many studies reported that the standard deviation and distribution of costs is likely to be skewed, we used the 1/3 of the average cost as the minimum value, and 3 times as the maximum to capture a broad plausible range.

**Model validation:**

Our simulations indicated a comparable expected life year between model prediction and the national life table among the female population (20.01 from model vs. 20.47 from life table), although a relatively higher expected life year was estimated from our model (19.17 vs 17.16). Additionally, we found that the 5-year event rate (except HF) was within reported ranges in two nationally representative Chinese cohorts: Chinese multi-provincial cohort study [[15](#_ENREF_15), [16](#_ENREF_16)] and China National Hypertension Survey and its follow up[[17-19](#_ENREF_17)] and some local cohorts[[20-22](#_ENREF_20)], such as data from Kailuan cohort study[[21](#_ENREF_21)]. Take MI for example, our simulation indicated that 1.25% of people would have an MI. This value falls within the reported rate varying from 0.19 to 1.50, which implies our model projected a reasonable estimate (see **Table E**).

| **Table A** Characteristics among the overall and those meeting SPRINT eligibility ^a^ | | |
| --- | --- | --- |
|  | Overall Sample | SPRINT Sample ^b^ |
| Sample, N | 13483 | 3000 |
| ***Socio-economic background*** |  |  |
| Age (years) |  |  |
| 45-49 | 2764(20.5%) | 0 |
| 50-59 | 4843(35.9%) | 851(28.4%) |
| 60-74 | 4803(35.6%) | 1579(52.6%) |
| ≥75 | 1073(8.0%) | 570(19.0%) |
| Educational level |  |  |
| None | 3843(28.5%) | 994(33.1%) |
| Primary | 5478(40.6%) | 1301(43.4%) |
| Secondary/High School | 3925(29.1%) | 654(21.8%) |
| College/University or above | 237(1.8%) | 51(1.7%) |
| Sex |  |  |
| Male | 6406(47.5%) | 1875(62.5%) |
| Female | 7077(52.5%) | 1125(37.5%) |
| ***Risk factors of hypertension*** |  |  |
| Heart rate, beats/min | 72.51±10.50 | 72.55±10.98 |
| Total cholesterol, mg/dl | 193.36±39.13 | 200.02±40.68 |
| High-density lipoprotein, mg/dl | 51.06±15.37 | 49.88±15.46 |
| Creatinine, mg/dl | 0.78±0.24 | 0.85±0.21 |
| Glomerular filtration rate, ml/min/1.73m^2^ | 91.66±16.86 | 84.64±16.48 |
| Currently smoking |  |  |
| Yes | 4170(30.9%) | 1296(43.2%) |
| No | 9313(69.1%) | 1704(56.8%) |
| BMI |  |  |
| BMI<25, kg/m^2^ | 9407(69.8%) | 2091(69.7%) |
| 25≤BMI<30 kg/m^2^ (Overweight) | 3402(25.2%) | 755(25.2%) |
| BMI≥30 kg/m^2^ (Obese) | 674(5.0%) | 154(5.1%) |
| Framingham risk score |  |  |
| <10% | 3994(41.4%) | 88(2.9%) |
| 10%-20% | 2743(28.4%) | 861(28.7%) |
| >20% | 2908(30.2%) | 2051(68.4%) |
| ***Blood pressure measurement*** |  |  |
| SBP |  |  |
| <130 mmHg | 7325(54.3%) | 0 |
| 130-139 mmHg | 2251(16.7%) | 959(32.0%) |
| ≥140 mmHg | 3907(29.0%) | 2041(68.0%) |
| DBP |  |  |
| <80 mmHg | 8735(64.8%) | 1247(41.6%) |
| 80-89 mmHg | 3062(22.7%) | 1084(36.1%) |
| ≥90 mmHg | 1686(12.5%) | 669(22.3%) |
| Anti-hypertension treatment |  |  |
| Untreated | 10920(81.0%) | 1994(66.5%) |
| Western modern medicine | 2232(16.5%) | 874(29.1%) |
| Chinese traditional medicine | 148(1.1%) | 62(2.1%) |
| Chinese and Western combined medicine | 183(1.4%) | 70(2.3%) |

Reported as Mean ± SD or number (%)

Abbreviation: DBP, Diastolic blood pressure. SBP, Systolic blood pressure. SPRINT, Systolic blood pressure intervention trial.

^a^ Missing values of total cholesterol, high-density lipoprotein and creatinine have been imputed by multi-imputation methods.

^b^ SPRINT include criteria: SBP 130-180 mm Hg; eGFR of 20-59 ml/min/1.73m^2^; 10-year risk for CVD≥15%; age≥75 years. Exclusion criteria: Diabetes, history of stroke, eGFR < 20 ml/min/1.73m^2^.

| **Table B** Numbers of overall participants and participants meeting each sequential SPRINT eligibility criterion, millions | | | | | |
| --- | --- | --- | --- | --- | --- |
|  | Overall | Age≥50y | +SBP criteria ^a^ | +High CVD risk ^b^ | +Exclusion Criteria ^c^ |
| Overall participants, N | 523.2 | 405.8 | 193.5 | 143.3 | 116.2 |
| ***Socio-economic background*** |  |  |  |  |  |
| Age (years) |  |  |  |  |  |
| 45-49 | 117.4(109.3-125.6) | 0 | 0 | 0 | 0 |
| 50-59 | 187.8(177.4-198.2) | 187.8(177.4-198.2) | 77.8(68.2-87.3) | 44.8(36.2-53.5) | 34.5(26.9-42.1) |
| 60-74 | 169.0(163.5-174.5) | 169.0(163.5-174.5) | 85.3(80.9-89.8) | 68.1(63.8-72.4) | 56.6(52.3-60.7) |
| ≥75 | 49.0(45.9-52.0) | 49.0(45.9-52.0) | 30.4(27.7-33.0) | 30.4(27.7-33.0) | 25.1(23.2-27.1) |
| Educational level |  |  |  |  |  |
| None | 133.2(125.8-140.7) | 121.9(114.5-129.3) | 61.2(54.3-68.2) | 41.7(35.5-47.9) | 35.8(29.5-41.9) |
| Primary | 202.8(194.4-211.2) | 164.1(157.2-171.1) | 79.8(73.7-85.9) | 62.4(56.4-68.3) | 49.9(45.6-54.3) |
| Secondary/High School | 173.3(164.6-181.9) | 110.9(104.5-117.3) | 48.2(42.7-53.6) | 36.2(31.3-41.2) | 28.0(23.6-32.4) |
| College/University or above | 13.9(10.2-17.6) | 8.9(6.9-10.8) | 4.3(2.5-6.2) | 3.0(2.2-3.7) | 2.5(1.8-3.2) |
| Sex |  |  |  |  |  |
| Female | 272.0(260.2-283.8) | 205.5(196.1-214.8) | 98.6(90.2-107.1) | 57.1(49.8-64.4) | 43.5(37.3-49.7) |
| Male | 251.2(242.6-259.9) | 200.3(192.5-208.1) | 94.9(88.0-101.8) | 86.1(79.6-92.7) | 72.7(66.7-78.7) |
| ***Risk factors of hypertension*** |  |  |  |  |  |
| Current smoking |  |  |  |  |  |
| Yes | 154.5(148.4-160.6) | 122.9(117.4-128.3) | 55.8(51.2-60.4) | 53.8(49.1-58.4) | 47.8(43.2-52.3) |
| No | 368.7(355.4-382.0) | 282.9(272.1-293.8) | 137.7(127.9-147.6) | 89.5(80.9-98.1) | 68.4(61.1-75.8) |
|  |  |  |  |  |  |
| BMI |  |  |  |  |  |
| BMI<25 kg/m^2^ | 351.4(342.2-360.7) | 278.7(271.5-285.8) | 120.3(114.5-126.0) | 91.8(86.4-97.2) | 78.8(73.9-83.6) |
| 25 ≤BMI<30 kg/m^2^ (Overweight) | 138.4(130.6-146.3) | 102.2(96.1-108.3) | 56.3(51.1-61.6) | 39.7(35.3-44.2) | 30.1(26.0-34.1) |
| BMI≥30 kg/m^2^ (Obese) | 33.4(25.2-41.6) | 24.9(17.2-32.6) | 16.9(9.3-24.5) | 11.7(4.4-19.0) | 7.3(1.0-13.7) |
| Framingham risk score |  |  |  |  |  |
| <10% | 213.7(204.5-222.7) | 124.6(119.3-129.8) | 26.2(23.1-29.4) | 3.1(2.1-4.2) | 3.0(2.0-4.1) |
| 10%-20% | 147.4(138.3-156.5) | 124.7(116.0-133.4) | 63.0(55.3-70.6) | 35.8(28.2-43.5) | 32.7(26.3-39.1) |
| >20% | 162.1(153.5-170.7) | 156.5(147.7-165.2) | 104.3(96.4-112.1) | 104.3(96.4-112.1) | 80.5(74.1-86.7) |
| ***Blood pressure measurement*** |  |  |  |  |  |
| SBP |  |  |  |  |  |
| <130 mmHg | 272.9(265.7-280.0) | 196.2(190.9-201.4) | 0 | 0 | 0 |
| 130-139 mmHg | 89.5(82.8-96.2) | 69.8(64.2-75.4) | 69.8(64.2-75.4) | 45.0(40.1-49.9) | 36.2(31.9-40.5) |
| ≥140 mmHg | 160.8(150.0-171.7) | 139.8(130.4-149.3) | 123.7(114.4-133.0) | 98.2(89.8-106.7) | 80.0(72.5-87.4) |
| DBP |  |  |  |  |  |
| <80 mmHg | 329.1(319.8-338.4) | 257.2(250.0-264.4) | 79.9(74.3-85.4) | 62.1(56.7-67.5) | 48.0(45.0-50.9) |
| 80-89 mmHg | 122.8(114.0-131.6) | 94.6(87.0-102.2) | 71.6(64.2-79.1) | 48.2(41.6-54.8) | 39.8(33.3-46.3) |
| ≥90 mmHg | 71.3(64.3-78.4) | 53.9(48.0-59.8) | 42.0(36.3-47.7) | 33.0(27.6-38.4) | 28.4(23.0-33.8) |
| Hypertension^d^ |  |  |  |  |  |
| Yes | 225.3(213.5-237.2) | 193.1(182.7-203.6) | 147.8(137.7-158.0) | 116.0(106.8-125.3) | 93.3(85.1-101.5) |
| No | 297.9(289.3-306.5) | 212.6(206.4-218.9) | 45.7(41.7-49.7) | 27.2(24.3-30.2) | 22.8(20.7-25.0) |
| Anti-hypertension treatment |  |  |  |  |  |
| Untreated | 418.4(405.7-431.2) | 313.4(302.8-324.0) | 130.5(121.2-139.8) | 87.7(79.6-95.8) | 77.2(69.3-85.0) |
| Western modern medicine | 90.1(84.0-96.2) | 79.0(74.4-83.5) | 53.3(49.2-57.5) | 46.5 (42.4-50.7) | 34.0(30.2-37.8) |
| Chinese traditional medicine | 8.2(4.4-12.0) | 7.4(3.6-11.2) | 5.7(1.9-9.5) | 5.3(1.5-9.2) | 2.6(1.9-3.2) |
| Chinese and Western combined medicine | 6.5(5.9-7.1) | 6.0(5.4-6.6) | 4(3.6-4.5) | 3.7(3.2-4.2) | 2.4(2.0-2.8) |

Reported as weighted numbers of subgroups (95% confidence interval);

Abbreviation: CVD, Cardiovascular disease. DBP, Diastolic blood pressure. SBP, Systolic blood pressure. SPRINT, Systolic blood pressure intervention trial;

^a^ SBP criteria include: 130-180 mm Hg;

^b^ High CVD risk include: history of CHD, eGFR of 20-59 ml/min/1.73m^2^, 10-year risk for CVD≥15%, and age≥75 years.

^c^ Exclusion criteria include: Diabetes, history of stroke, eGFR < 20 ml/min/1.73m^2^.

^d^ Defined as SBP≥140 mm Hg or DBP≥90 mm Hg or taking antihypertensive medication.

| **Table C** Probability of new events after disease and treatment effect from intensive BP treatment | | | |
| --- | --- | --- | --- |
| **Description** | **Value (range)** | **Distributions** | **Reference** |
| **MI** |  |  |  |
| MI incidence | Exponential (age, BP treatment, SBP, TC, HDL-C, WC, DM, Smoking, Geographic region, Urbanization, Family history of ASCVD) | Uniform | [[23](#_ENREF_23)] |
| Heart failure during the first year after MI | 0.121 | Beta | [[24](#_ENREF_24)] |
| Stroke during the first year after MI | 0.031 | Beta | [[24](#_ENREF_24)] |
| Reinfarction during the first year after MI | 0.067 | Beta | [[24](#_ENREF_24)] |
| Long-term heart failure after MI | 0.038 | Beta | [[24](#_ENREF_24)] |
| Long-term stroke after MI | 0.019 | Beta | [[24](#_ENREF_24)] |
| Long-term reinfarction after MI | 0.014 | Beta | [[24](#_ENREF_24)] |
| MI mortality | First year: 0.057; beyond: SMR 1.43(95%CI:1.18–1.72) on background mortality | Beta/ Lognormal | [[24](#_ENREF_24), [25](#_ENREF_25)] |
| **Stroke** |  |  |  |
| Stroke incidence | Exponential (age, BP treatment, SBP, TC, HDL-C, WC, DM, Smoking, Geographic region, Urbanization, Family history of ASCVD) | Uniform | [[23](#_ENREF_23)] |
| MI first year after Stroke | 0.0167 | Beta | [[26](#_ENREF_26)] |
| Recurrent during the first year after Stroke | 0.164 for men and 0.1988 for women | Beta | [[27](#_ENREF_27)] |
| Long-term recurrent Stroke after Stroke | 0.0998 | Beta | [[28](#_ENREF_28)] |
| Long-term MI year after Stroke | 0.012 | Beta | [[28](#_ENREF_28)] |
| Stroke mortality | First year: 0.123 for male, 0.178 for female; beyond: SMR 2.23(95%CI:1.29–3.88) on background mortality | Beta/ Lognormal | [[25](#_ENREF_25), [27](#_ENREF_27)] |
| **HF** |  |  |  |
| HF incidence | Exponential (age, sex, SBP, DM, LVH, BMI, heart rate, CHD, valve disease) | Uniform | [[29](#_ENREF_29)] |
| Stroke during the first year after HF | 0.0184 | Beta | [[30](#_ENREF_30)] |
| Long-term stroke during the first year after HF | 0.0121 | Beta | [[30](#_ENREF_30)] |
| HF mortality | First year: 0.159; beyond: 0.08 | Beta | [[31-33](#_ENREF_31)] |
| **Serious Adverse event** |  |  |  |
| SAE rate | 0.0288(0.0001-0.0330) | Triangle distribution | ^[^[^3-5^](#_ENREF_3)^,^ [^8^](#_ENREF_8)^]^ |
| **Mortality** |  |  |  |
| All-cause mortality | Age and sex dependent | Uniform | [[34](#_ENREF_34)] |
| **Treatment effect** |  |  |  |
| MI | 0.83(95%CI:0.64-1.09) | Lognormal | ^[^[^35^](#_ENREF_35)^]^ |
| HF | 0.62(95%CI:0.45-0.84) | Lognormal | ^[^[^35^](#_ENREF_35)^]^ |
| Stroke | 0.89(95%CI:0.63,1.25) | Lognormal | ^[^[^35^](#_ENREF_35)^]^ |
| CVD mortality | 0.57(95%CI:0.38,0.85) | Lognormal | ^[^[^35^](#_ENREF_35)^]^ |
| All- cause mortality | 0.73(95%CI:0.60-0.90) | Lognormal | ^[^[^35^](#_ENREF_35)^]^ |
| Serious Adverse event | 1.43 (1.27,1.70) | Triangle distribution | ^[^[^4^](#_ENREF_4)^],[^[^8^](#_ENREF_8)^]^ |

| **Table D** Annual event cost and Quality of life weight | | | |
| --- | --- | --- | --- |
| **Description** | **value** | **Distributions** | **Reference** |
| **Cost^a^** |  |  |  |
| Annual cost for intensive blood pressure treatment | $642($389-$1089) | Gamma | **[**[**6**](#_ENREF_6)**]** |
| Annual cost for standard blood pressure treatment | $385($243-$714) | Gamma | [[6](#_ENREF_6)] |
| Annual cost for Stroke | $5914($1971-$17742) first year, post $2545($848-$7635) | Gamma | [[10](#_ENREF_10), [11](#_ENREF_11), [34](#_ENREF_34)] |
| Annual cost for MI | $13254($4418-$39762) first year, post $739($246-$2217) | Gamma | [[12](#_ENREF_12), [34](#_ENREF_34)] |
| Annual cost for HF | $2853($951-$8559) first year, post $589($196-$1767) | Gamma | [[14](#_ENREF_14), [34](#_ENREF_34)] |
| Annual cost for SAE | $1687($562-$5061) | Gamma | [[5](#_ENREF_5)] |
| **Quality of life weights(utilities)** |  |  |  |
| No cardiovascular disease | Age and sex dependent | Uniform | [[36](#_ENREF_36)] |
| Stroke | 0.63 (0.26 to 0.92) | Beta | [[37](#_ENREF_37)] |
| MI | First year: 0.76 (0.5 to 0.87); beyond: 0.88 (0.67 to 0.94) | Beta | [[37](#_ENREF_37)] |
| HF | 0.71 (0.43 to 0.84) | Beta | [[37](#_ENREF_37)] |
| AE | 0.50 for the first week; beyond: utility back to respective event. | Uniform | [[4](#_ENREF_4)] |
| **Discount rate** | 3% (0%-5%) | Uniform | Assumed |

^a^ To convert cost input to Chinese currency, multiply by purchasing power parity (PPP) rate (in this case, 3.54).

MI: Myocardial Infarction; HF: Heart Failure; SAE: Serious Adverse Event

| **Table E** Comparison of the 5-year incidences or life-years with published data | | |
| --- | --- | --- |
|  | **Predicted estimates (95% CI) from our microsimulation model** | **Published data among Chinese population** |
| MI (%) | 1.25(1.03, 1.47) | 0.19-1.50[[15-18](#_ENREF_15), [20](#_ENREF_20), [21](#_ENREF_21)] |
| Stroke (%) | 2.28(1.99,2.57) | 0.33-2.61[[15-18](#_ENREF_15), [20](#_ENREF_20), [21](#_ENREF_21)] |
| HF (%) | 2.06(1.78, 2.34) | No available data |
| CVD mortality (%) | 4.34(3.94, 4.74) | 5.25[[19](#_ENREF_19)] 5.93[[22](#_ENREF_22)] |
| All-cause mortality (%) | 11.48(10.86, 12.10) | 11.23[[19](#_ENREF_19)] 15.63[[22](#_ENREF_22)] |
| Expected life years at mean age | 20.01(Female) 19.17(Male) | 20.47(Female);17.16 (Male) ^a^ |

^a^ Calculated based on life tables of Chinese population in 2017 at age 65.

**Table F** 5 year and life-time events difference for different outcomes from base-case analysis

|  |  | **Intensive BP**  **control** | **Standard treatment** | **Difference** | **Number of events saved in million** |
| --- | --- | --- | --- | --- | --- |
| MI | 5-year | 0.01190  (0.0098,0.01403) | 0.01250  (0.01032,0.01468) | -0.0006  (-0.0036,0.0024),p=0.2986 | 0.069  (-0.28, 0.42) |
|  |  |  |  |  |  |
|  | Life-time | 0.04510  (0.04103,0.04917) | 0.04820  (0.04400,0.05240) | -0.0031  (-0.0089, 0.0027),p=0.6991 | 0.36  (-0.31, 1.03) |
|  |  |  |  |  |  |
| Stroke | 5-year | 0.01970  (0.01698,0.02242) | 0.02280  (0.01987,0.02573) | -0.0031  (-0.0071,0.0009), p=0.5515 | 0.36  (-0.10,0.82) |
|  |  |  |  |  |  |
|  | Life-time | 0.1054  (0.09938,0.1114) | 0.1080  (0.1019,0.1141) | -0.0026  (-0.0112, 0.0060), p=0.1285 | 0.30  (-0.70, 1.30) |
|  |  |  |  |  |  |
| HF | 5-year | 0.01340  (0.0115,0.01565) | 0.02060  (0.01782,0.2338) | -0.0072  (-0.0108, -0.0036),p<0.0001 | 0.84  (0.42,1.25) |
|  |  |  |  |  |  |
|  | Life-time | 0.04650  (0.04237,0.05063) | 0.0620  (0.05727,0.6673) | -0.0155  (-0.0218, -0.0092),p<0.0001 | 1.80  (1.07, 2.53) |
|  |  |  |  |  |  |
| CVD mortality | 5-year | 0.02590 (0.02279, 0.02901) | 0.04340 (0.03941,0.04739) | -0.0175  (-0.0226, -0.0124),p<0.0001 | 2.03  (1.44, 2.63) |
|  |  |  |  |  |  |
|  | Life-time | 0.1844  (0.1768,0.1920) | 0.2485  (0.2400,0.2570) | -0.0641  (-0.0755, -0.0527), p<0.0001 | 7.45  (6.12, 8.77) |
|  |  |  |  |  |  |
| Years of Life | 5-year | 4.8130  (4.7990,4.8270) | 4.7791  (4.7639,4.7943) | 0.0339(0.0132,0.546),p<0.0001 | 3.84  (1.53, 6.34) |
|  | Life-time | 20.6480  (20.4240,20.8720) | 19.6955  (19.4727,19.9183) | 0.9525(0.6366,1.2684),p<0.0001 | 11.06  (9.74, 14.73) |

**Table G** Total costs of intensive BP control and standard BP treatment

|  | **Intensive BP control ^a^** | **Standard BP treatment ^a^** | **Difference ^b^** |
| --- | --- | --- | --- |
| Antihypertension treatment costs | 8690 | 4960 | 4039(395,8744) |
| Cost of complications | 1774 | 2280 | -570(-751,181) |
| SAE costs | 931 | 612 | 308(257,359) |
| Total costs | 11395 | 7861 | 3777(-208,8286) |

^a^ To convert cost input to Chinese currency, the cost was multiplied by purchasing power parity (PPP) rate (in this case, 3.54). Total cost includes antihypertension treatment costs, cost of complications and serious adverse event costs.

^b^  Probabilistic analyses will run the model 1000 times with the use of randomly selected values for input measurements from predefined distributions. The uncertainty intervals (UIs) show the 2.5 to 97.5 percentiles for the incremental differences in costs.

**Reference:**

1. Zhao YS, J; Yang, G; Giles J; Hu P; Hu Y; Lei X; Park A; Smith JP; Wang Y. China Health and Retirement Longitudinal Study-2011–2012.National Baseline Users Guide.2013.

2. Tao C, Yu DH, Cornelius V, Rui Q, Cai YM, Jiang ZX, et al. Potential health impact and cost-effectiveness of drug therapy for prehypertension. Int J Cardiol. 2017;240:403-8. doi: 10.1016/j.ijcard.2017.05.003. PubMed PMID: WOS:000405454800073.

3. Law MR, Wald NJ, Morris JK, Jordan RE. Value of low dose combination treatment with blood pressure lowering drugs: analysis of 354 randomised trials. BMJ. 2003;326(7404):1427. Epub 2003/06/28. doi: 10.1136/bmj.326.7404.1427. PubMed PMID: 12829555; PubMed Central PMCID: PMCPMC162261.

4. Richman IB, Fairley M, Jorgensen ME, Schuler A, Owens DK, Goldhaber-Fiebert JD. Cost-effectiveness of Intensive Blood Pressure Management. Jama Cardiol. 2016;1(8):872-9. doi: 10.1001/jamacardio.2016.3517. PubMed PMID: WOS:000401861300006.

5. Gu DF, He J, Coxson PG, Rasmussen PW, Huang C, Thanataveerat A, et al. The Cost-Effectiveness of Low-Cost Essential Antihypertensive Medicines for Hypertension Control in China: A Modelling Study. Plos Medicine. 2015;12(8). doi: ARTN e1001860

10.1371/journal.pmed.1001860. PubMed PMID: WOS:000360708300006.

6. Su M, Zhang Q, Bai X, Wu C, Li Y, Mossialos E, et al. Availability, cost, and prescription patterns of antihypertensive medications in primary health care in China: a nationwide cross-sectional survey. Lancet. 2017;390(10112):2559-68. Epub 2017/11/06. doi: 10.1016/S0140-6736(17)32476-5. PubMed PMID: 29102087.

7. Joint Committee for Guideline R. 2018 Chinese Guidelines for Prevention and Treatment of Hypertension-A report of the Revision Committee of Chinese Guidelines for Prevention and Treatment of Hypertension. J Geriatr Cardiol. 2019;16(3):182-241. doi: 10.11909/j.issn.1671-5411.2019.03.014. PubMed PMID: 31080465; PubMed Central PMCID: PMCPMC6500570

8. Bress AP, Bellows BK, King JB, Hess R, Beddhu S, Zhang Z, et al. Cost-Effectiveness of Intensive versus Standard Blood-Pressure Control. N Engl J Med. 2017;377(8):745-55. Epub 2017/08/24. doi: 10.1056/NEJMsa1616035. PubMed PMID: 28834469; PubMed Central PMCID: PMCPMC5708850.

9. Wang Z, Chen Z, Zhang L, Wang X, Hao G, Zhang Z, et al. Status of Hypertension in China: Results From the China Hypertension Survey, 2012-2015. Circulation. 2018;137(22):2344-56. Epub 2018/02/17. doi: 10.1161/CIRCULATIONAHA.117.032380. PubMed PMID: 29449338.

10. Wang Y, Li L, Ma W. Factor analysis of costs of hospitalized patients with stroke who participated in urban and rural medical insurance in Tianjin. Chinese Journal of Hospital Statistics. 2014;21(6):401-4.

11. Yuanmei P, Yansheng L, Yan L, Ruifang W, Min S, Xuan H, et al. Study on drug cost burdens of standard secondary prevention for ischemic st roke patients in Shanghai. Shanghai Med J 2010;33(9):808-13.

12. Wu Y, Li M, Xuan J, Zelt S, Yin H, Zhou Q, et al. A Cost-Effectiveness Analysis between Amlodipine and Angiotensin Ii Receptor Blockers in Stroke and Myocardial Infarction Prevention among Hypertension Patients in China. Value in Health. 2012;15(7):A631-A. PubMed PMID: WOS:000312411102019.

13. Yuan S, Li N, Zhang Y, Gu Q, Liu Y, Ma J. Effect of different health coverage on hospital expdenditure of patients with acute myocardial infarction Journal of Shanghai Jiaotong University(Mecial Science). 2013;33(2):214-9.

14. Yu SB, Zhao QY, Cui HY, Qin M, Liu T, Kong B, et al. [Investigation on the prevalence and related factors of medicinal therapy in patients with chronic systolic heart failure]. Zhonghua Liu Xing Bing Xue Za Zhi. 2012;33(2):229-33. Epub 2012/05/12. PubMed PMID: 22575150.

15. Liu J, Hong YL, D'Agostino RB, Wu ZS, Wang W, Wu GX, et al. The predictive value for Chinese population using the Framingham CHD risk assessment tool and the model derived from the chinese multi-provincial cohort study. Circulation. 2003;108(17):753-. PubMed PMID: WOS:000186360603452.

16. Wei W, Dong Z, jing L, Guixian w, Zhechun z, Liu. J, et al. Prospective study on the predictive model of cardiovascular disease risk in a Chinese population aged 35-64. Chin J Cardiol. 2003;31(12):902-8.

17. Wu YF, Liu XQ, Li X, Li Y, Zhao LC, Chen Z, et al. Estimation of 10-year risk of fatal and nonfatal ischemic cardiovascular diseases in Chinese adults. Circulation. 2006;114(21):2217-25. doi: 10.1161/Circulationaha.105.607499. PubMed PMID: WOS:000243406300007.

18. Gu DF, Kelly TN, Wu XG, Chen J, Duan XF, Huang JF, et al. Blood pressure and risk of cardiovascular disease in chinese men and women. American Journal of Hypertension. 2008;21(3):265-72. doi: 10.1038/ajh.2007.59. PubMed PMID: WOS:000253323000010.

19. He J, Gu D, Chen J, Wu X, Kelly TN, Huang JF, et al. Premature deaths attributable to blood pressure in China: a prospective cohort study. Lancet. 2009;374(9703):1765-72. Epub 2009/10/09. doi: 10.1016/S0140-6736(09)61199-5. PubMed PMID: 19811816.

20. Zhang XF, Attia J, D'Este C, Yu XH, Wu XG. A risk score predicted coronary heart disease and stroke in a Chinese cohort. Journal of Clinical Epidemiology. 2005;58(9):951-8. doi: 10.1016/j.jclinepi.2005.01.013. PubMed PMID: WOS:000231543200012.

21. Wu SL, Huang ZR, Yang XC, Li SQ, Zhao HY, Ruan CY, et al. Cardiovascular events in a prehypertensive Chinese population: Four-year follow-up study. Int J Cardiol. 2013;167(5):2196-9. doi: 10.1016/j.ijcard.2012.05.123. PubMed PMID: WOS:000323569600093.

22. Wang JB, Huang QC, Hu SC, Zheng PW, Shen P, Li D, et al. Baseline and longitudinal change in blood pressure and mortality in a Chinese cohort. J Epidemiol Community Health. 2018;72(12):1083-90. Epub 2018/08/06. doi: 10.1136/jech-2018-211050. PubMed PMID: 30077965.

23. Yang X, Li J, Hu D, Chen J, Li Y, Huang J, et al. Predicting the 10-Year Risks of Atherosclerotic Cardiovascular Disease in Chinese Population: The China-PAR Project (Prediction for ASCVD Risk in China). Circulation. 2016;134(19):1430-40. Epub 2016/09/30. doi: 10.1161/CIRCULATIONAHA.116.022367. PubMed PMID: 27682885.

24. Liang Y, Zhu J, Tan HQ, Zhang Y, Liu LS, Chinese Coordinating Center of OR. [Risk factors associated with increased end points of patients with non-ST elevation acute coronary syndromes in China: 2 years follow up results of China-OASIS Registry]. Zhonghua Xin Xue Guan Bing Za Zhi. 2009;37(7):580-4. PubMed PMID: 19961723.

25. Sai XY, He Y, Men K, Wang B, Huang JY, Shi QL, et al. All-cause mortality and risk factors in a cohort of retired military male veterans, Xian, China: an 18-year follow up study. Bmc Public Health. 2007;7. doi: Artn 290

10.1186/1471-2458-7-290. PubMed PMID: WOS:000252727700001.

26. Boulanger M, Bejot Y, Rothwell PM, Touze E. Long-Term Risk of Myocardial Infarction Compared to Recurrent Stroke After Transient Ischemic Attack and Ischemic Stroke: Systematic Review and Meta-Analysis. J Am Heart Assoc. 2018;7(2). Epub 2018/01/20. doi: 10.1161/JAHA.117.007267. PubMed PMID: 29348322; PubMed Central PMCID: PMCPMC5850155.

27. Wang Z, Li JJ, Wang CX, Yao XM, Zhao XQ, Wang YL, et al. Gender Differences in 1-Year Clinical Characteristics and Outcomes after Stroke: Results from the China National Stroke Registry. Plos One. 2013;8(2). doi: ARTN e56459

10.1371/journal.pone.0056459. PubMed PMID: WOS:000315970300164.

28. Wong KS, Li H. Long-term mortality and recurrent stroke risk among Chinese stroke patients with predominant intracranial atherosclerosis. Stroke. 2003;34(10):2361-6. doi: 10.1161/01.Str.0000089017.90037.7a. PubMed PMID: WOS:000185679100027.

29. Kannell WB, D'Agostino RB, Silbershatz H, Belanger AJ, Wilson PWF, Levy D. Profile for estimating risk of heart failure. Archives of Internal Medicine. 1999;159(11):1197-204. doi: DOI 10.1001/archinte.159.11.1197. PubMed PMID: WOS:000080823800007.

30. Witt BJ, Gami AS, Ballman KV, Brown RD, Meverden RA, Jacobsen SJ, et al. The incidence of ischemic stroke in chronic heart failure: A meta-analysis. Journal of Cardiac Failure. 2007;13(6):489-96. doi: 10.1016/j.cardfail.2007.01.009. PubMed PMID: WOS:000257502300011.

31. Hua YH, Wu NQ, Lu XF, Xie GQ, Zhang J, Gu DF, et al. [Body mass index and prognosis in patients with systolic heart failure]. Zhonghua Xin Xue Guan Bing Za Zhi. 2009;37(10):870-4. PubMed PMID: 20137533.

32. Yu S, Zhao Q, Cui H, Qin M, Liu T, Kong B, et al. Long-term prognostic value of atrial fibrillation in patients with chronic systolic heart failure. Chin J Cardiac Arrhyth. 2012;16(1):39-45.

33. Sun l, Lv R, Liang T, Ji S, Kang X, Guo J, et al. Prognosis status with its influencing factors in heart failure patients during 1 year of dischaged period Chinese Circulation Journal 2013;25(2):125-8.

34. National Bureau of Statistics of the People's Republic of China. China statistical yearbook. Beijing(CN): China Statistics Press. 2018.

35. Group SR, Wright JT, Jr., Williamson JD, Whelton PK, Snyder JK, Sink KM, et al. A Randomized Trial of Intensive versus Standard Blood-Pressure Control. N Engl J Med. 2015;373(22):2103-16. doi: 10.1056/NEJMoa1511939. PubMed PMID: 26551272; PubMed Central PMCID: PMCPMC4689591.

36. Sun S, Chen JY, Johannesson M, Kind P, Xu L, Zhang YG, et al. Population health status in China: EQ-5D results, by age, sex and socio-economic status, from the National Health Services Survey 2008. Quality of Life Research. 2011;20(3):309-20. doi: 10.1007/s11136-010-9762-x. PubMed PMID: WOS:000288214800002.

37. Geisler BP, Egan BM, Cohen JT, Garner AM, Akehurst RL, Esler MD, et al. Cost-Effectiveness and Clinical Effectiveness of Catheter-Based Renal Denervation for Resistant Hypertension. Journal of the American College of Cardiology. 2012;60(14):1271-7. doi: 10.1016/j.jacc.2012.07.029. PubMed PMID: WOS:000309508400011.
