# Supplementary material for: Applicability and cost-effectiveness of the Systolic Blood Pressure Intervention Trial (SPRINT) in the Chinese population: A cost-effectiveness modeling study
Source: PLoS Med. 2021 Mar 4;18(3):e1003515. doi: 10.1371/journal.pmed.1003515 (PMC7971845; doi:10.1371/journal.pmed.1003515)
Supplement: Attachment — Submitted filename: Point-by-point responses PMEDICINE-D-20-01125R1_24 Jun 2020.docx [file pmed.1003515.s007.docx]

**Requests from the editors:**

 ***Please revise your title according to PLOS Medicine's style - this should be structured with the main study question and then the study design (eg, : cost-effectiveness modelling) after a colon in the subtitle.**

*Response: Thank you for this suggestion, we have now added the subtitle of “**cost-effectiveness modelling” as suggested.*
 ***At this stage, we ask that you include a short, non-technical Author Summary of your research to make findings accessible to a wide audience that includes both scientists and non-scientists. The Author Summary should immediately follow the Abstract in your revised manuscript. This text is subject to editorial change and should be distinct from the scientific abstract. Please see our author guidelines for more information:** [**https://journals.plos.org/plosmedicine/s/revising-your-manuscript#loc-author-summary**](https://protect-eu.mimecast.com/s/eHLiCqlqnU5LGjtl-MJF?domain=journals.plos.org)

*Response: We have included this Author Summary on [page 5 line 98-121] as below:*

*“Why was this study done?*

- *The Systolic Blood Pressure Intervention Trial (SPRINT) has previously demonstrated significant reductions in death and cardiovascular disease (CVD) risk with a systolic blood pressure (SBP) goal of < 120 mmHg (intensive treatment) compared with a SBP goal of < 140 mmHg (standard treatment)*
- *A large proportion of Chinese adults are classified as hypertensive but few of them achieve recommended blood pressure targets*

*What did the researchers do and find?*

- *We used nationally representative data from the China Health and Retirement Longitudinal Study (CHARLS) (2011-12) to assess the applicability of the SPRINT trial to the Chinese adult population, and a validated microsimulation model to predict the cost-effectiveness of this intensive treatment among those meeting the SPRINT eligibility*
- *It is estimated that 116.2 million adults in China are eligible for the SPRINT intensive treatment strategy*
- *If adopted, intensive treatment has the potential to prevent 2.03 million cardiovascular disease deaths with a potential gain of 3.84 million more life-years over five years and would likely be cost-effective over a lifetime*

*What do these findings mean?*

- *A substantial number of Chinese adults meet SPRINT eligibility criteria*
- *This evidence suggests that intensive treatment among high-risk populations could be worthy of significant capital investment*

*“*

***In the last sentence of the Abstract Methods and Findings section, please note any key limitation(s) of the study's methodology. The authors might also want to include a summary in the abstract of the outcomes from any of the sensitivity analyses, not just the primary analyses, in the study as this will help readers grasp how robust the findings are to underlying assumptions used in the models.**

*Response: Thank you for your comment. We have now added the following text to Methods and Findings section of Abstract on [page 4 line 77-81]:*

*“Main limitations include lack of specific SPRINT eligibility information in the CHARLS survey, uncertainty about the implications from the different BP measurement, the use of several sources of data with large reliance on findings from SPPRINT, limited information about SAE rate, lack of information and evidence for medication effectiveness on renal disease.”*

*We have also added the following text regarding our sensitivity analyses to the Methods and Findings section of Abstract on [page 4 line 71-77] as follows:*

*“Over a lifetime, intensive treatment increased the mean QALYs from 9.51 to 9.87 (an increment of 0.38 [95%CI:0.13-0.71]) and would cost international $10,997 per QALY gained over standard treatment.* *Of all one-way sensitivity analyses, high antihypertensive drug cost, lower treatment efficacy to CVD death resulted in the most two unfavourable results (Int$25,291 and Int $18,995 per QALY were gained, respectively). Simulation results indicated that intensive treatment could be cost-effective (82.8% below the willingness-to-pay threshold of international $16,782 [1*GDP per capita in China in 2017]) with a lower probability in subjects with SBP 130-139 mmHg (72.9%) but a higher chance among female population(91.2%). ”*

***If possible, please modify the intext reference callouts to use numbers in square brackets (eg [1, 2]) - if referencing software is used this should be fairly quick and easy.**

*Response: We have changed the format of the reference as suggested.*

**Comments from the reviewers:**

**Reviewer #1: The propsed paper is well written and with a well done statistical analysis modeling. Furthermore conclusions are driven by founded results. However some revision is needed before it could be accepted for pubblication. The mail problem is how blood pressure was measured in the study from which data were taken for the model. In fact, SPRINT trial was hardly criticized by european reserchers and guidelines since measurements were done** **by unattended automated BP device. If BP in the CHARLS study has been taken differently (whit classic methods) these data could not be compared with the SPRINT one. In fact,** **unattended BP results in 10-15 mmHg less BP and so the main criticism is that probably SPRINT is comparing actual normal control (130 mmHg) with uncontrolled BP (150 mmHg) so only confirming the actual guidelines and not confirming the intensive treatment benefit. Please better describe methods for BP measurements and discuss this issue.**

*Response: Many thanks for your point. BP was measured three times (approximately 45s apart) by* *an attended automated BP device (Omron model HEM-7200) in CHARLS study. Obviously, this is different from the method used in the SPRINT trial as mentioned, although the SPRINT protocol did not particularly address the issue of staff attendance during the 5-minute rest period and/or during the time the Omron was automatically taking the BP measurements. We have added the following text to describe the BP measurement in the Appendix in page 1 under the section of ‘Study variables and definition’: “BP was taken by attended automated BP device (Omron model HEM-7200). We calculated the average SBP and DBP based on 3 blood pressure measurements (**approximately 45s apart) for each subject.”*

*We agree with your point and believe that this difference may have affected our results. We included your point in the limitation on [page 17 line 375-382] as below:*

*“Fourthly, BP measurements in SPRINT were likely done by unattended automated BP device, which are different from the one used in CHARLS with staff attendance during BP measurement. Studies have shown that unattended BP can result in 10-15 mmHg less BP. This may have affected the proportion of subjects meeting SPRINT criteria and our further modelling results. That said, evidence has shown a similar BP level and cardiovascular disease risk reduction in the intensive group in SPRINT participants, regardless of the measurement technique [28].”*

**Furthermore, I din't understand why in the "base-case analysis" section authors reports only on the reduction of HF incidence and CV fatal events while not considering non fatal events. Please better describe.**

*Response: Thanks for your comment. We have changed the description to include the non-fatal event on [page 13 line 286-295]as below:*

*“Compared to standard treatment, intensive SBP treatment was predicted to reduce the incidence rate within 5 years of HF by 0.72% (95% CI: 0.36%, 1.08%) , MI 0.06% (95% CI: -0.24, 0.36%) , stroke 0.31% (95% CI: 0.09%, 0.71%) and CVD mortality 1.75% (95% CI: 1.24%, 2.26%). Correspondingly, we estimate the 5-year events reduction would be 0.84 million (95% CI: 0.42, 1.25) for HF, 0.069 million (95% CI: -0.28, 0.42) for MI, 0.36 million (95% CI: -0.10, 0.82) for stroke and 2.03 million (95% CI: 1.44, 2.63) for CVD mortality. Taken together, 3.84 million (95% CI: 1.53, 6.34) life-years could be saved from intensive SBP treatment compared to standard treatment within five years, and 11.06 million (95% CI: 9.7, 14.73) life-years over a lifetime (Figure 3, S6 Table).”*

**Reviewer #2: The manuscript concerns a very important topic of intensifying blood pressure treatment, which is a cheap and effective option that may affect a large proportion of population. However, I have a number of questions regarding the modelling assumptions:

- Which treatment effects on cardiovascular endpoints were used in the model? eTable 3 suggests these were the hazard effects coming directly from the SPRINT trial (which is exactly what I would expect). But then what is depicted in eFigure2? Also text, lines 199-201.**

*Response: Thank you for your points. Yes, you are right. The treatment effect here refers to the hazard ratios (HR) in the SPRINT trial. The published HR in the eFigure 2 is exactly the HRs reported from the SPRINT study. The purpose of eFigure2 is to show whether our model can replicate the treatment effect (i.e. HR) as the SPRINT trial within a short time period. Meanwhile, we compared the event rates (e.g., MI or HF rate) derived from our simulation model with the reported data from those who receiving standard treatment under current guideline.*

*To avoid confusion, we changed the title of eFigure 2 as: “**Hazard Ratios from the simulation modelling within 5 year comparing with published HRs in the SPRINT study”*

*Also, the words in the main text on [page 12 line 280] were changed to “Secondly, we also computed the predicted 5-year CHD, stroke and HF incidences and mortality rates among subjects with standard treatment from our base-case model and compared these with published data.”*

**- Adverse events: why is the HR of 1.43 used if SPRINT reported 1.04? Further, why is a SAE assumed to incur annual costs for the lifetime duration but the QoL decrement is assumed to be back to normal after one week? Where would the costs come from then?**

*Response: We captured the HR of 1.43 for adverse event from the cost-effectiveness analysis of the SPRINT study. It can be identified from Table 1 from that publication (Bress AP, Bellows BK, King JB, et al. Cost-Effectiveness of Intensive versus Standard Blood-Pressure Control. N Engl J Med. 2017;377(8):745-755). We also varied the uncertainty broadly after taking into account another published paper (Richman IB, Fairley M, Jorgensen ME, Schuler A, Owens DK, Goldhaber-Fiebert JD. Cost-effectiveness of Intensive Blood Pressure Management. Jama Cardiol. 2016;1(8):872-879.).*

*We ran our model using 12-month cycles. Cost could be added if SAEs were captured during each annual cycle. However, we assumed that the quality-of-life for people with SAEs (e.g., hypotension) would be back to normal status quickly if they recovered, rather than remaining in a chronic status as other states, such as stroke or HF. We followed the same procedure as another studies and made some discounts for the QoL once a SAE was found.*

*Regarding the source of the SAE cost, it is from page 9 Table 1 in another cost-effectiveness analysis in China after inflating to 2017 (Gu DF, He J, Coxson PG, et al. The Cost-Effectiveness of Low-Cost Essential Antihypertensive Medicines for Hypertension Control in China: A Modelling Study. Plos Medicine. 2015;12(8)).*

*All references mentioned above have already been cited in the* *Table A4.*

**- Why is it assumed that further CV events may only happen within the next year of the first one?**

*Response: We completely agree with your point and in fact we have included the secondary event (e.g., Stroke after MI) within the first year of event for each disease path. This detailed description can be found in the appendix page 2 under the section ‘Model Structure’.*

**- In terms of the proportion of people not treated for hypertension, is it possible to use re-interview data, as presumably the situation has changed wince 2011.**

*Response: Thanks for your suggestion. We agree that the prevalence of untreated hypertension could change during the follow up of this cohort. However, our study was not aiming to investigate the dynamic changes on this prevalence, which have been discussed in many other studies.*

*We tried to assess the impact of the SPRINT trial to the Chinese population using nationally representative data. The number of individuals without hypertension treatment was estimated after accounting for the sample weight of CHARLS study, which was given at baseline survey. Also, we believe that using the re-interview data, rather than baseline data, would change the sampling frame (eg, death or lost follow-up) and result in inaccurate estimates.*

**- eTable 5: could the authors add description of the population / any other assumptions used in the comparator table, to make it clearer that like-with-like is being compared.**

*Response: Many thanks for your suggestion. We have added one section in the Appendix to detail this.*

*“Model validation*

*Our simulations indicated a comparable expected life year between model prediction and the national life table among the female population (20.01 from model vs. 20.47 from life table), although a relatively higher expected life year was estimated from our model (19.17 vs 17.16). Additionally, we found that the 5-year event rate (except HF) was within reported ranges in Chinese population. Take MI for example, our simulation indicated that 1.25% of people would have an MI. This value falls within the reported rate varying from 0.19 to 1.50, which implies our model projected a reasonable estimate (see eTable 5). While comparing the model derived HR with the published HR in the SPRINT trial, we could identify that the estimated HRs are similar or within published HR, which means our simulation was calibrated to the treatment effect in Chinese population (eFigure 2). “*

**- One ICER may mask important differences in population subgroups. Could the results be presented by age groups and categories of blood pressure.**
*Response: Thank you for your advice. Since we did not have the individual data, it was not possible for us to derive the treatment effects by age and blood pressure and incorporate them into our model. However, our study has presented the result by individual subgroups (i.e. age, blood pressure and gender) in Table 1 while the treatment effects are available from the publication of the SPRINT trial.*

**I am also struggling to understand information presented in Figure 3. What scale is it presented on? The x-axis suggests it may be on a person-level, but how can 7 deaths be averted for one person? In general, information on lifetime number of deaths avoided is not necessarily meaningful - people will have to die eventually; besides those that live longer, may well experience CV events just as much as those who live less; it's just they experience them later. Perhaps something like "numbers needed to treat to avoid 1 stroke in 5 years" would be more meaningful. Related to this, in eTable 6, the numbers of strokes saved over lifetime is smaller than that saved over 5-years, with the trend reversed with every other endpoint. There may well be a reason for this, but it should be noted and discussed.**

*Response: Thanks for your points. We have now amended to make this clearer. It is at the scale of the whole Chinese population after employing the sample weights. We have changed the label for the y-axis as “Events averted (million)”. Also, the legend for this figure was changed as per suggestions of the third reviewer on [page 21 line 536-540] as below.*

*“Figure 3: Base case 5 year and life-time events averted for different outcomes*

*The ’events averted’ refers to events that that would be avoided if the intensive treatment were in place instead of standard treatment. It is calculated by multiplying the event different from our simulation with the number of SPRINT eligible adults.*

*MI=Myocardial infarction; HF=Heart failure; CVD=Cardiovascular disease”*

*We agree that the information on the lifetime number of events may be confounded by the death benefits from the treatment. This competing risk between the event and death would be stronger for the long-term estimates comparing with the short-term ones. Meanwhile, the small and uncertain treatment benefits for Stroke or MI were reported in the SPRINT trial. These two points may explain the issues mentioned. We have added some text to notify this limitation of our estimates on [page 17 line 382-384] as below:*

*“Finally, our estimates of the events averted are likely confounded by the competing risk between event and death, particularly for the life-time estimates”*

**A few minor comments:**
**- Table 1: costs and ICERs should be rounded to the nearest integer, if not more; also it should be clarified what "incremental costs" refers to - total costs or just hospital costs? Ir the former, it would be very helpful to see specifically hospital costs. Treatment costs could be deduced from life expectancy & treatment cost.**

*Response: Thanks for your correction. We have corrected them in Table 1 and the associated figure in the text.*

*The incremental costs refer to the total cost from the model. The hospital cost for each event was extracted from China Health Statistics Yearbook 2018, which can be found in the appendix under the section of Cost.*

**- There were quite a few spelling errors, particularly in the appendix, could this be reviewed carefully.**

*Response: Thanks for highlighting this. We have been over the text carefully and believe that we have now corrected all such errors.*

**Reviewer #3: This is a statistical review of manuscript PMEDICINE-D-20-01125R1. It is well structured, well written and the topic is of great importance. From a statistical viewpoint, I would like to see 2 main clarifications:**

**1/ Line 236 : Our model showed a reasonable concordance between model predicted risk estimates and published hazard ratios (HRs) (0.86 vs.0.83 for MI, 0.95 vs. 0.89 for stroke, 0.65 vs. 0.62 for HF, 0.59 vs. 0.57 for CVD mortality, 0.85 vs. 0.73 for all-cause mortality). What are these HRs ? Are they the ratios of the hazards of MI, Stroke etc between patients on intensive treatment vs standard?**

*Response: Yes. They are the hazard ratios from the SPRINT study. To avoid any confusion, we have added the following text on [page 12 line 280] as below:*

*“Our model showed a reasonable concordance between model predicted risk estimates and published hazard ratios (HRs) from the SPRINT study (0.86 vs.0.83 for MI, 0.95 vs. 0.89 for stroke, 0.65 vs. 0.62 for HF, 0.59 vs. 0.57 for CVD mortality, 0.85 vs. 0.73 for all-cause mortality)”*

**Figure S2 should explain in the legend what HRs are plotted**

*Response: Many thanks for your point. We have changed the legend as below: “Published HR from the SPRINT study” and changed the figure title as “Hazard Ratios from the simulation modelling within 5 year comparing with published HRs in the SPRINT study”*

**2/ Figure 3: what is the scale of the Y-axis? Is it at the level on an individual, or is it at the scale of the whole Chinese population (based on the text line 251 this is probably the case)? Some CIs reach the negative territory - does it mean that the intensive treatment may increase MI and stroke? I think that the legend should make clear that these are the events that would be avoided if the intensive treatment was in place instead of the standard one.**

*Response: Yes, you are right. It is at the scale of the whole Chinese population after employing the sample weights. We have changed the title of this figure and added some words in the legend as per suggestions of the second reviewer on [page 21 line 536-540] as below:*

*“Figure 3: Base case 5 year and life-time events averted for different outcomes*

*‘Events averted’ refers to events that that would have been avoided had the intensive treatment been in place instead of the standard one. It was calculated by multiplying the event different from our simulation with the number of SPRINT eligible adults*

*MI=Myocardial infarction; HF=Heart failure; CVD=Cardiovascular disease”*

*Regarding the negative values in MI and stroke, we believe this is corresponding to the treatment effect of the SPRINT study, where the lower CI is less than 1. As our model highly relied on this information, our estimates would reflect the uncertainty. This limitation was already included in the main text.*

**Minor points: in some instances, there are sentences where words are missing or the English could be improved. For example:
"Although adoption of the SPRINT treatment strategy would increase the number of Chinese adults requiring SBP [treatment?] intensification"**

*Response: Thanks for your point. We have now corrected this.*
 **"this approach have [has?] the"**

*Response: Many thanks for your correction. We have now changed this.*

**"the detailed disease states within each path could be [can be found?] in our previous publication"**
*Response: Many thanks for your correction. We have made the corresponding revision.*
