# Supplementary material for: Applicability and cost-effectiveness of the Systolic Blood Pressure Intervention Trial (SPRINT) in the Chinese population: A cost-effectiveness modeling study
Source: PLoS Med. 2021 Mar 4;18(3):e1003515. doi: 10.1371/journal.pmed.1003515 (PMC7971845; doi:10.1371/journal.pmed.1003515)
Supplement: Attachment — Submitted filename: Point-by-point responses PMEDICINE-D-20-01125R2_11 Sep 2020.docx [file pmed.1003515.s008.docx]

**Requests from the editors:**

1- **Thank you for editing your title. Please edit again slightly: “Applicability and Cost-effectiveness of the Systolic Blood Pressure Intervention Trial (SPRINT) in the Chinese Population: a Cost-effectiveness modelling study”**

*Response: Thank you for this suggestion, we have now added the subtitle of “**a cost-effectiveness modelling study” as suggested.* **2- Thank you for adding an Author Summary. Please edit the last point of the Author Summary to: “This evidence suggests that intensive treatment to reducing blood pressure among high-risk populations could be cost effective.” (or something similar).**

*Response: Thank you for this suggestion, we have now edited the last point of the Author Summary as suggested.*

**3- Throughout the manuscript, please include the p-values alongside 95% CIs when relevant.**

*Response: Many thanks for your points. we added the p value in S6 Table where inferential tests are applied. however, for others, such as the prevalence estimation in figure 2, p values are not provided as there are no inferential tests performed. For estimates in Table 1, probabilistic analyses were used to assess the uncertainty of our estimates from each model parameter. This method is different from the common inferential tests used in other study design such as trial or observational studies. Therefore, no P values are provided.*

**4- Thank you for adding the non-fatal events in response to reviewer 1’s second point. It does look like for MI and stroke, the reductions are not significant. Please make this clear by separating these outcomes out in the text.**

*Response: Thank you for your point. we have reworded it as below:*

*Result on [page 13 line 288-294]*

*“Compared to standard treatment, intensive SBP treatment was predicted to reduce the incidence rate within 5 years of HF by 0.72% (95% CI: 0.36%, 1.08%) , and CVD mortality 1.75% (95% CI: 1.24%, 2.26%) , though not significantly for MI 0.06% (95% CI: -0.24, 0.36%) , stroke 0.31% (95% CI: 0.09%, 0.71%). Correspondingly, we estimate the 5-year events reduction would be 0.84 million (95% CI: 0.42, 1.25) for HF, 2.03 million (95% CI: 1.44, 2.63) for CVD mortality, 0.069 million (95% CI: -0.28, 0.42) for MI and 0.36 million (95% CI: -0.10, 0.82) for stroke.”*

*Meanwhile, we edited the Abstract on [page 3 line 66-69] accordingly.*

*“**We estimated that over a five-year horizon, compared to standard treatment, intensive treatment would reduce 0.84 (95%CI:0.42-1.25) million heart failure incidences, 0.069 million (95% CI: -0.28, 0.42) myocardial infarction, 0.36 million (95% CI: -0.10, 0.82) stroke and 2.03 (95%CI:1.44-2.63) million CVD deaths, respectively, and save 3.84 (95%CI:1.53-6.34) million life-years.”*

**5- Please include the url and any accession numbers necessary to apply for access to the CHARLS dataset.**

*Response: CHARLS data could be accessible to researchers around the world at the study website(**http://charls.pku.edu.cn/index/en.html). We followed the protocol and applied it after registration. The data could be downloaded after approval using our registered account. There are no accession numbers needed.*

*We now added the URL in the text on [page 7 line 158-159]*

*“ This public data could be accessible via study website (*<http://charls.pku.edu.cn/index/en.html>*).”*

**6- Did your study have a prospective protocol or analysis plan? Please state this (either way) early in the Methods section.

a) If a prospective analysis plan (from your funding proposal, IRB or other ethics committee submission, study protocol, or other planning document written before analyzing the data) was used in designing the study, please include the relevant prospectively written document with your revised manuscript as a Supporting Information file to be published alongside your study, and cite it in the Methods section. A legend for this file should be included at the end of your manuscript.

b) If no such document exists, please make sure that the Methods section transparently describes when analyses were planned, and when/why any data-driven changes to analyses took place.

c) In either case, changes in the analysis-- including those made in response to peer review comments-- should be identified as such in the Methods section of the paper, with rationale.**

*Response: I am sorry that we did not have a prospective protocol or analysis plan written for this study particularly. However, the analysis of this study was based on our previous published model, which included a 22- page technical report in the appendix and was cited in our current work. In addition to this main cost-effectiveness analysis, we also present the number of subject eligible for SPRINT treatment, which is a simple and straightway analysis. We describe the method in the main text, detailed the associated part and included the results in our 20-page appendix.*

*Our previous published work is listed as below.*

[*Tao Chen*](https://pubmed.ncbi.nlm.nih.gov/?term=Chen+T&cauthor_id=28501349)*, [Dahai Yu](https://pubmed.ncbi.nlm.nih.gov/?term=Yu+D&cauthor_id=28501349),* [*Victoria Cornelius*](https://pubmed.ncbi.nlm.nih.gov/?term=Cornelius+V&cauthor_id=28501349)*,* [*Rui Qin*](https://pubmed.ncbi.nlm.nih.gov/?term=Qin+R&cauthor_id=28501349) *, [Yamei Cai](https://pubmed.ncbi.nlm.nih.gov/?term=Cai+Y&cauthor_id=28501349) , [Zhixin Jiang](https://pubmed.ncbi.nlm.nih.gov/?term=Jiang+Z&cauthor_id=28501349) , [Zhanzheng Zhao](https://pubmed.ncbi.nlm.nih.gov/?term=Zhao+Z&cauthor_id=28501349). Potential health impact and cost-effectiveness of drug therapy for prehypertension. Int J Cardiol. 2017;240:403-408.*

**Comments from the reviewers:

Reviewer #2: Thank you for providing answers to my comments. I would still like to have a few points (from my original comments) clarified, and it would be helpful if these were clarified in the manuscript text as well.

* Treatment effect. Thank you for clarifying these were the SPRINT treatment effects that were used in the modelling. I would then suggest removing S2 Figure from the manuscript as it does not add anything. The simulated HRs are not simulated, but are taken from literature, so of course they will be the same when plotted against the HRs they are equal to. The small difference presumably comes from projecting subsequent events, population sampling and randomness in the simulation process.**

*Response: Thank you for your point, we generally agreed with your idea, however, we believe this figure could still provide some information for our model validation, although it is limited.*

*In our previous published model, we employ two methods to compare the event rate and life year with the reported data among Chinese population. In addition to this, our current model followed two recently published models in NEMJ (please see page 49 in the appendix) and JAMA cardiology (please see figure 2) to further compare the modeled HRs or event rate with the published ones. This is to show our model could generally maintain the inputted HRs even incorporating the uncertainty from various model parameters and randomness in the simulation process.*

*The mentioned two papers are listed below:*

*Bress AP, Bellows BK, King JB, Hess R, Beddhu S, Zhang Z, et al. Cost-Effectiveness of Intensive versus Standard Blood-Pressure Control. N Engl J Med. 2017;377(8):745-55*

*Richman IB, Fairley M, Jorgensen ME, Schuler A, Owens DK, Goldhaber-Fiebert JD. Cost-effectiveness of Intensive Blood Pressure Management. Jama Cardiol. 2016;1(8):872-9.*

*** Cost of SAEs. So if an SAE has occurred, will the costs be** **applied every single year after it occurred (despite QoL being back no normal within a week) or only** **in a year that it occurred?**

*Response: Cost of SAEs could only be included in the year when it occurred. This is different from other disease status, such as stroke. Further annual cost for rehabitation is needed after an acute event.*

*To clarify this, we reworded the description for cost of SAEs as below on [page 10 line 228-230]:*

*“For the cost of SAEs, it was from a publication but was only applied in the year when it occurred.”*

*** Modelling subsequent CV events. I understand that secondary events may happen, but why can they only happen in the year after the primary event? Whilst the risk of a subsequent event is higher in the next year, it does not go away in years after that**

*Response: Thank you very much for your point. we are sorry that we did not clarify it well. we now changed the S1 Fig to detail the disease pathway and rewrote the description for model structure to avoid using the words of the secondary events (i.e., event within the year after primary event). Meanwhile, we included the* *schematic depiction for each event for your information. Since they are from our published work, we could not include them in our current appendix, but we cited this work. Also, we do acknowledge the higher risk after acute event (eg, MI) and already included this in our model, which was already mentioned on page 9 line 199-201 and could be identified from the S3 Table in the appendix.*

*A new detailed S1 Fig are indicated below on [page 13] in the appendix.*

*Schematic depiction for each event in our previous work is listed as below.*

*The reworded description for model structure in the appendix is shown as below on [ page 2]:*

*“For each individual disease path, patients may either survive or die from a disease suffered within a period. During the first year, patients might experience more than one event (e.g. the occurrence of stroke after MI). After an event, patients transitioned into one of the following chronic health states: post MI, post stroke and post HF. While being in these states, patients might suffer from further CVD events. Schematic depictions for each event could be found in appendix of our previous work[**2**].”*

*** Proportion of people on hypertension. This comment referred to the authors' estimation that 2/3 of those meeting SPRINT eligibility criteria were not treated for hypertension (line 336, page 15; also abstract). However, this was the case in 2011, since then most likely this number has decreased. I assume that in the "standard treatment" scenario everyone was prescribed antihypertensive medications rather than just those that were recorded to do so. Could the authors confirm this please.**

*Response: This cost-effectiveness analysis is based on a mathematical simulation, which compared two treatment strategies proposed in SPRINT study among the same target population (here is the SPRINT eligible subjects). That means everyone who are eligible for the SPRINT study will be given antihypertensive treatment, regardless of their BP treatment history. This is in line with the SPRINT study and will maintain the comparability of the two treatments.*

*** eTable5: The added text just re-phrases the results that are presented. it still not clear that the populations for which results are presented (ie population in this study vs populations in references 30-37) are comparable. Could this be commented on please.**

*Response: Thank you for your comments. Model validation is always a problem as there is no standard way to follow. however, this part is required in the ISPOR guidelines. eTable 5 is part of the result for the model validation/calibration, which aimed to show the estimates from our model, such as the incidence rate of MI, were within the range of data from representative Chinese hypertensive population.*

*References 30-37 are selected because they are mainly from two cohorts:* *Chinese multi-provincial cohort study and China National Hypertension Survey and its follow up. The population of these two cohorts are nationally representative sample of the general population with long-term follow up. thus, we believe their results could best reflect the event rate among Chinese hypertensive population. Meanwhile, we also cited three contemporary local cohorts with large sample size and median term follow up to capture the uncertainty of the event rate as time goes.*

*We edited the text to include the description about the population in previous references 30-37 (now updated to 15-22) on [page 4]*

*“Additionally we found that the 5-year event rate (except HF) was within reported ranges in two nationally representative Chinese cohorts: Chinese multi-provincial cohort study [**15**,* *16**] and China National Hypertension Survey and its follow up[**17-19**] and some local cohorts[**20-22**], such as data from Kailuan cohort study[**21**] “*

*** Figure 3. What exactly does it mean to say that 7 million CVDs were averted over lifetime? Does it mean that these 7 million people died of non-CVD causes instead? It seems misleading to present this number unless there is some discussion in the text.**

*Response: Thank you very much for sharing your concerns. We would not interpret the averted 7 million CVDs as 7 million death from non-CVDs because any treatment can not change the fact that human has to die for some causes in the end. However, treatment may alter the disease pathway. For example, a person may experience MI at 50 and died from it. However, he/she could have MI later (let us say at 60 years old) and died from by accidence at 70 if successful intervention was adopted. As demonstrated from our model, we can tell that the treatment could change the proportion of CVD death, which is often the claiming benefits from BP treatment.*

*We agree with your points that presenting this number may mislead our readers. Meanwhile, we do acknowledge this issue from competing risk from non-CVD mortality and would like to remind our readers to interpret the data with great cautions, particularly for our lifetime estimates.*

*We now changed the text in the limitation on [page 17 line 383-387].*

*“Finally, although BP intensification, like many other interventions, could not avoid the death of subjects in the end, it may alter their disease progress or pathway. Therefore, our estimates on the events or deaths averted may be confounded by the competing risk between event and CVD death* *or non-CVD death, particularly for the life-time estimates.”*

*** Comment on Table 1: It is still unclear what costs are presented in column named "Costs, Int $". Are these hospital costs, or do they include treatment costs? Regardless, it would be very helpful to look separately at hospital costs and treatment costs.**

*Response: Many thanks for your points. the cost in the Table 1 is the total cost which includes the hospital costs and disease treatment costs. As described in our text, the cost information is mainly from the national health statistics report, in which no detailed cost components are provided. Therefore, we are sorry that we could not provide the cost by type and we agree that it is helpful to readers who are interested in this. However, cost analysis by type is not our planned objective, and we believed our current analysis could answer our question: what is the cost-effectiveness of two BP treatment strategy?*

*** Spelling: there are still some spelling mistakes (eg Figure 2: "adverted" should be "averted")**

*Response: Thank you very much for your correction. We corrected it.*
