# Supplementary material for: Applicability and cost-effectiveness of the Systolic Blood Pressure Intervention Trial (SPRINT) in the Chinese population: A cost-effectiveness modeling study
Source: PLoS Med. 2021 Mar 4;18(3):e1003515. doi: 10.1371/journal.pmed.1003515 (PMC7971845; doi:10.1371/journal.pmed.1003515)
Supplement: Attachment — Submitted filename: revision.docx [file pmed.1003515.s009.docx]

Thank you for your comments. Unfortunately most of the comments were still not address, and of those that were, the clarifications were not reflected in the text. Could the authors please add clarifications in the text as well as reviewers comments. I will now copy-paste my comments from the previous review, some of which appeared in the first review already, and reply to the authors’ replies.

1. **Original comment**

Treatment effect. Thank you for clarifying these were the SPRINT treatment effects that were used in the modelling. I would then suggest removing S2 Figure from the manuscript

as it does not add anything. The simulated HRs are not simulated, but are taken from

literature, so of course they will be the same when plotted against the HRs they are equal

to. The small difference presumably comes from projecting subsequent events, population

sampling and randomness in the simulation process.

**Revision 3 comment.** HRs derived from the same data they were obtained from originally are expected to be the same, so presenting S2 Figure does not add anything to the manuscript. It certainly does not confirm the model validity. All it does is saying that a measure derived in the same way from the same dataset is the same. If the authors decide to leave this figure in, this point needs to be made very clear in the text. Additionally, I don’t understand the following comment from the authors’ reply:

- In addition to this, our current model followed two recently published models in NEMJ (please see page 49 in the appendix). and JAMA cardiology (please see figure 2) to further compare the modeled HRs or event rate with the published ones.

Firstly, the appendix attached to the paper only has 20 pages, so there is no page 49. Secondly, the validation section does not contain reference to Figure 2. If the authors would like to compare the HRs from these models, could they quantify the comparison in the Model validation section.

2. **Original comment**

Modelling subsequent CV events. I understand that secondary events may happen, but why can they only happen in the year after the primary event? Whilst the risk of a subsequent event is higher in the next year, it does not go away in years after that

**Revision 3 comment**

Thank you for the reply. The following point is still unclear. Could the authors please address them both in the replies as well as the text.

The main text states that “. At the end of every model cycle (i.e., one year), an individual will either transit to another health state or remain in the same state, with the probabilities sampled from defined distributions. Subsequent to a primary event (i.e., myocardial infarction [MI], heart failure [HF], or stroke), patients move to a chronic heath state in which they have a higher risk of suffering a further CVD event or death.” From here I conclude that no more than one event can happen in any given year, that a subsequent event may happen at any point after the first event, and that any CV event event may happen after any CV event (eg repeated events are allowed).

However, the Appendix says that “During the first year, patients might experience more than one event (e.g. the occurrence of Stroke after MI).” What does the ‘first year’ mean? First year after the first event? First year of simulation? Same year as the year of the event? Is this what “the first year after” / “long-term” probabilities in S3 Table refer to? Could this be made clear, and consistent, in the text.

Further, S1 Figure suggests that no repeated events are modelled, eg MI cannot happen after the MI. This is a legitimate assumption but should be clarified in the text. S3 Table however suggests that repeated events may happen, but then S1 Figure should reflect this.

3. **Original comment**

Proportion of people on hypertension. This comment referred to the authors' estimation that 2/3 of those meeting SPRINT eligibility criteria were not treated for hypertension (line 336, page 15; also abstract). However, this was the case in 2011, since then most likely this number has decreased. I assume that in the "standard treatment" scenario everyone was prescribed antihypertensive medications rather than just those that were recorded to do so. Could the authors confirm this please.

**Revision 3 comment**

Could the authors please respond (both here and in the text) to the first part of the comment, regarding the estimation of the proportion of people not on treatment.

Also, thank you for confirming what “standard treatment” was; could this be clarified in the text as well please

4. **Original comment**

eTable5: The added text just re-phrases the results that are presented. it still not clear that the populations for which results are presented (ie population in this study vs populations in references 30-37) are comparable. Could this be commented on please.

**Revision 3 comment**

What I meant in my question is whether the estimates in Column 2 (model predicted estimates (95% CI)) are based on applying the model to the population from the references in Column 3, or are they the estimates obtained from applying the model to the SPRINT population. Could the authors clarify this in the text, and also comment specifically on the similarities (and differences) between the SPRINT population and that in Column 3.

5. **Original comment**

Figure 3. What exactly does it mean to say that 7 million CVDs were averted over lifetime? Does it mean that these 7 million people died of non-CVD causes instead? It seems misleading to present this number unless there is some discussion in the text.

**Revision 3 comment**

I still don’t understand how the number of lifetime CVD mortality events was calculated. The legend says “The ’events averted’ refers to events that that would be avoided if the intensive 548 treatment were in place instead of standard treatment. It is calculated by multiplying the event different from our simulation with the number of SPRINT eligible adults”. To me it reads as if these people did not experience CVD death. Why would this not mean that these people experienced non-CVD death, which is what the authors’ reply suggests? What other possible outcomes are there? Could the authors please provide the methodology, as well as the explanation both in the reply as well as an in the text, including the Figure 3 legend, because as it stands, this is a misleading message.

6. **Original comment**

Comment on Table 1: It is still unclear what costs are presented in column named "Costs, Int $". Are these hospital costs, or do they include treatment costs? Regardless, it would be very helpful to look separately at hospital costs and treatment costs.

**Revision 3 coment**

Could the authors please incorporate their answers (ie explanation of what costs are presented) into the manuscript text, including the Table caption or legend. Secondly, while I appreciate that cost information on specific diagnosis codes may be difficult to obtain, splitting the costs by total treatment costs vs total event costs should be very easy to do from the model – indeed, that is how total costs are usually calculated. It would provide a necessary context for the reader to understand what the cost differences are driven by, which, in turn would help place results in a general context (ie what happens if treatment intervention price changes etc)
