# Supplementary material for: Applicability and cost-effectiveness of the Systolic Blood Pressure Intervention Trial (SPRINT) in the Chinese population: A cost-effectiveness modeling study
Source: PLoS Med. 2021 Mar 4;18(3):e1003515. doi: 10.1371/journal.pmed.1003515 (PMC7971845; doi:10.1371/journal.pmed.1003515)
Supplement: Attachment — Submitted filename: Point-by-point responses PMEDICINE-D-20-01125R3_14Dec 2020.docx [file pmed.1003515.s010.docx]

**Requests from Editors:**

**1- Please address the remaining comments from reviewer 2 and incorporate your responses into the main text (not just the rebuttal letter), in case readers are also unclear.**

*Response: Thank you for this suggestion, we have now addressed the comments from reviewer 2 and revised our main text accordingly, which can be found in our response below.*

**2- Please report your economic analysis according to the appropriate study design provided at** [**http://www.equator-network.org/?post_type=eq_guidelines&eq_guidelines_study_design=economic-evaluations&eq_guidelines_clinical_specialty=0&eq_guidelines_report_section=0&s=**](https://protect-eu.mimecast.com/s/00_HCNYgBS0X47OI7wI7n?domain=equator-network.org) **and provide the relevant completed checklist. In the checklist please include sufficient text excerpted from the manuscript to explain how you accomplished all applicable items.
Please add the following statement, or similar, to the Methods: "This study is reported as per the XXX guideline (S1 Checklist)."
When completing the checklist, please use section and paragraph numbers, rather than page numbers.**

*Response: We have now added the checklist in the appendix and filled the checklist following the instruction using section and paragraph numbers. Please find the S1 Table CHEERS checklist.* **3- Thank you for updating your data statement. However, I note that the webpage you direct to reads: “All data will be made public one year after the end of data collection.” PLOS requires that the data underlying all analyses in the paper be available at the time of publication. Can you confirm that the data are available now? If so, update the data statement to read: “CHARLS data are available via the study website: ” and provide a more specific url to direct readers to the dataset. If the dataset is not currently available, please let us know the date when it will be available.
It seems that this paper uses data from wave 1 and 2 (2011-2012), which seem to be available
here:**
**[http://charls.pku.edu.cn/pages/data/2011-charls-wave1/en.html](https://protect-eu.mimecast.com/s/PwMaCOPjDUp4Wn7hpAvyk?domain=charls.pku.edu.cn)
and here:**
[**http://charls.pku.edu.cn/pages/data/2012-charls-pilot-wave2/en.html**](https://protect-eu.mimecast.com/s/8PDACPzkEiKMpWjIP8rzJ?domain=charls.pku.edu.cn) **If that is correct, please use these urls in the data statement.**

*Response: Yes. the CHARLS data up to wave 4 in 2018 are available to users now. In this study, we only use the baseline survey (wave 1), which was conducted between June 2011 and March 2012.*

*We revised the data statement as below on [page 7 line 167-168] :*

*“CHARLS data are available via the website:* [*http://charls.pku.edu.cn/pages/data/2011-charls-wave1/en.html*](https://protect-eu.mimecast.com/s/PwMaCOPjDUp4Wn7hpAvyk?domain=charls.pku.edu.cn)*”*

**4- Line 65: remove “horizon”: “We estimated that over five years…”**

*Response: Thank you for this suggestion, we have changed it accordingly.* **5- Thank you for editing the language around MI and stroke. However, please further edit the line in the abstract to reflect that the 95% CIs do not predict a reduction in MI or stroke incidence. I suggest moving these outcomes to a separate sentence, e.g., “****Estimated reductions of 0.069 million (95% CI: -0.28, 0.42) myocardial infarction and 0.36 million (95% CI: -0.10, 0.82) stroke incidences were not statistically significant.”**

*Response: Thanks for your suggestion. We have revised the text as per your advice on [page 3 line 68-70]:*

*“We estimated that over five years, compared to standard treatment, intensive treatment would reduce 0.84 (95%CI:0.42-1.25) million heart failure incidences, 2.03 (95% CI:1.44-2.63) million CVD deaths, and save 3.84 (95%CI:1.53-6.34) million life-years. Estimated reductions of 0.069 million (95% CI: -0.28, 0.42) myocardial infarction and 0.36 million (95% CI: -0.10, 0.82) stroke incidences were not statistically significant.”*

**6- Thank you for your response regarding the planned analyses and your previous published model. Please note this in the methods section and cite the study.**

*Response: We have added a paragraph to describe this as instructed on [page 7 line 154-159].*

*“This study is composed of two parts: population analysis and health economic modelling. It was planned at the time of study conception on this basis of our previous work[13], although no formal prospective analysis plan was recorded. A further analysis of splitting the total cost by its components was added in response to peer reviewer’s comments (see S8 Table). This study is reported as per the Consolidated Health Economic Evaluation Reporting Standards (CHEERS) guideline (S1 Table).”*

**7- At lines 56 and 157, “... aged over 45 *years*...”**

*Response: Thank you for corrections. We have corrected them.* **8- Line 77 and throughout, please replace “subjects” with “people”.**

*Response: Thank you for this suggestion, we have replaced “subjects” with “people” as suggested.* **9- Throughout the manuscript, please replace the abbreviation “y” with “years”.**

*Response: Thank you for corrections. We have replaced “y” with “years” throughout the manuscript.*
 **10- Please remove “As a landmark study in hypertension management,” from line 388.**

*Response: Thank you for this suggestion, we have now deleted it as suggested.*

**11- Please include information on ethics approval and consent obtained for the CHARLS study when mentioning it in the Methods section.**

*Response: We added the below information in the Method as required on [page 7,8 line 173-175]:*

*“**The ethics committee of Peking University Health Science Center approved this study, and all participants gave written informed consent before participation.”*

**12- The supporting information, please replace “gender” with “sex”.**

*Response: Many thanks for your points. We have replaced “gender” with “sex” as suggested.*

**Thank you for your comments. Unfortunately most of the comments were still not address, and of those that were, the clarifications were not reflected in the text. Could the authors please add clarifications in the text as well as reviewers comments. I will now copy-paste my comments from the previous review, some of which appeared in the first review already, and reply to the authors’ replies.**

1. **Original comment**

**Treatment effect. Thank you for clarifying these were the SPRINT treatment effects that were used in the modelling. I would then suggest removing S2 Figure from the manuscript as it does not add anything. The simulated HRs are not simulated, but are taken from literature, so of course they will be the same when plotted against the HRs they are equal to. The small difference presumably comes from projecting subsequent events, population sampling and randomness in the simulation process.**

**Revision 3 comment. HRs derived from the same data they were obtained from originally are expected to be the same, so presenting S2 Figure does not add anything to the manuscript. It certainly does not confirm the model validity. All it does is saying that a measure derived in the same way from the same dataset is the same. If the authors decide to leave this figure in, this point needs to be made very clear in the text. Additionally, I don’t understand the following comment from the authors’ reply:**

- **In addition to this, our current model followed two recently published models in NEMJ (please see page 49 in the appendix). and JAMA cardiology (please see figure 2) to further compare the modeled HRs or event rate with the published ones.**

**Firstly, the appendix attached to the paper only has 20 pages, so there is no page 49. Secondly, the validation section does not contain reference to Figure 2. If the authors would like to compare the HRs from these models, could they quantify the comparison in the Model validation section.**

*Response:*

*Many thanks for your point. We are sorry that we could not clarify this and mislead you.*

*To avoid confusion, we accepted your advice and deleted S2 Figure.*

*For the model predicted HR, this is calculated by comparing the risk of events under two study treatments on the basis of our simulation population. Concisely, they are from our microsimulation model based on CHARLS data in China. However, for the published HRs from the SPRINT study, they are from the SPRINT study in the US. This means the HRs in the S2 Figure are calculated from two different sources.*

*The reason of presenting this figure is to show that our constructed model itself could maintain the inputted parameters given that the randomness could exist in the model simulation process. This idea comes from the mentioned two papers (please see below) and we just employed their approach to validate our model. We are sorry that we did not cite it again in our responses.*

*The two papers and their model validation could be found below:*

1, Bress AP, Bellows BK, King JB, Hess R, Beddhu S, Zhang Z, Berlowitz DR, Conroy MB, Fine L, Oparil S, Morisky DE, Kazis LE, Ruiz-Negron N, Powell J, Tamariz L, Whittle J, Wright JT, Jr., Supiano MA, Cheung AK, Weintraub WS, Moran AE, Group SR. Cost-Effectiveness of Intensive versus Standard Blood-Pressure Control. *N Engl J Med.* 2017;377:745-755.

2, Richman IB, Fairley M, Jorgensen ME, Schuler A, Owens DK, Goldhaber-Fiebert JD. Cost-effectiveness of Intensive Blood Pressure Management. *Jama Cardiol.* 2016;1:872-879.

2. **Original comment**

**Modelling subsequent CV events. I understand that secondary events may happen, but why can they only happen in the year after the primary event? Whilst the risk of a subsequent event is higher in the next year, it does not go away in years after that**

**Revision 3 comment**

**Thank you for the reply. The following point is still unclear. Could the authors please address them both in the replies as well as the text.**

**The main text states that “. At the end of every model cycle (i.e., one year), an individual will either transit to another health state or remain in the same state, with the probabilities sampled from defined distributions. Subsequent to a primary event (i.e., myocardial infarction [MI], heart failure [HF], or stroke), patients move to a chronic heath state in which they have a higher risk of suffering a further CVD event or death.” From here I conclude that no more than one event can happen in any given year, that a subsequent event may happen at any point after the first event, and that any CV event event may happen after any CV event (eg repeated events are allowed).**

**However, the Appendix says that “During the first year, patients might experience more than one event (e.g. the occurrence of Stroke after MI).” What does the ‘first year’ mean? First year after the first event? First year of simulation? Same year as the year of the event? Is this what “the first year after” / “long-term” probabilities in S3 Table refer to? Could this be made clear, and consistent, in the text.**

**Further, S1 Figure suggests that no repeated events are modelled, eg MI cannot happen after the MI. This is a legitimate assumption but should be clarified in the text. S3 Table however suggests that repeated events may happen, but then S1 Figure should reflect this.**

*Response:*

*We appreciate your point and your interpretation on the main text is correct. It is our carelessness to separate this model description into two places. What we want to say in the appendix is that our model considers the possibility of having another event within the same year after the first event. For example, a patient may have MI but he/she could have stroke during that year. This is what “the first year after” / “long-term” probabilities in S3(now S4) Table refer to.*

*We now put these two together in the main text as below on [page 9 line 213-215]:*

*“Subsequent to a primary event (i.e., myocardial infarction [MI], heart failure [HF], or stroke), patients move to a chronic heath state in which they have a higher risk of suffering a further CVD event or death. Also, our model includes the disease pathway of experiencing more than one event (e.g. the occurrence of Stroke after MI) during the first year of primary event.”*

*We agreed that our S1 Figure could not detail the model structure, thus we replace it with the following figure, which can both show the pathway after the first event and the recurrence of event.*

3. **Original comment**

**Proportion of people on hypertension. This comment referred to the authors' estimation that 2/3 of those meeting SPRINT eligibility criteria were not treated for hypertension (line 336, page 15; also abstract). However, this was the case in 2011, since then most likely this number has decreased. I assume that in the "standard treatment" scenario everyone was prescribed antihypertensive medications rather than just those that were recorded to do so. Could the authors confirm this please.**

**Revision 3 comment**

**Could the authors please respond (both here and in the text) to the first part of the comment, regarding the estimation of the proportion of people not on treatment.**

**Also, thank you for confirming what “standard treatment” was; could this be clarified in the text as well please**

*Response: Many thanks for your points and we agree with your point that our estimates for the proportion of people on hypertension may be altered with time. We add this point in our limitation to remind our readers. Please find the revised sentence below on [page 18 line 393-396].*

*“Fifth, our estimations on the number or proportion of Chinese adults meeting SPRINT eligibility criteria are based on survey data at one time point (i.e., 2011-2012), which may be changed with the time, particularly for those with or without hypertension treatment.”*

*Meanwhile, we added the texts to clarify the treatment used in our simulation on [page 9 line 199-200]:*

*“We developed a microsimulation model to project health-care costs, clinical outcomes, and quality-adjusted life-years (QALYs) among the SPRINT-eligible Chinese adults for antihypertensive treatment (regardless of their BP treatment history) under two alternative SBP treatment strategies:”*

4. **Original comment**

**eTable5: The added text just re-phrases the results that are presented. it still not clear that the populations for which results are presented (ie population in this study vs populations in references 30-37) are comparable. Could this be commented on please.**

**Revision 3 comment**

**What I meant in my question is whether the estimates in Column 2 (model predicted estimates (95% CI)) are based on applying the model to the population from the references in Column 3, or are they the estimates obtained from applying the model to the SPRINT population. Could the authors clarify this in the text, and also comment specifically on the similarities (and differences) between the SPRINT population and that in Column 3.**

*Response: Many thanks for clarifying your question.*

*The estimates in column 2 are from our microsimulation model based on CHARLS data in China, which are used to compare the data reported among Chinese in Column 3 for model validation purpose.*

*The main objective of our study is to test what happen if SPRINT study was adopted in China. therefore, model validation in our study is mainly to show that our constructed model could generally reflect the nature history of hypertension among Chinese rather than SPRINT study population from America.*

*To avoid confusion, we change the column head title of S5 table (now S6 table) as Predicted estimates (95% CI) from our microsimulation model (column 2) and Published data among Chinese population (column 3).*

5. **Original comment**

**Figure 3. What exactly does it mean to say that 7 million CVDs were averted over lifetime? Does it mean that these 7 million people died of non-CVD causes instead? It seems misleading to present this number unless there is some discussion in the text.**

**Revision 3 comment**

**I still don’t understand how the number of lifetime CVD mortality events was calculated. The legend says “The ’events averted’ refers to events that that would be avoided if the intensive 548 treatment were in place instead of standard treatment. It is calculated by multiplying the event different from our simulation with the number of SPRINT eligible adults”. To me it reads as if these people did not experience CVD death. Why would this not mean that these people experienced non-CVD death, which is what the authors’ reply suggests? What other possible outcomes are there? Could the authors please provide the methodology, as well as the explanation both in the reply as well as an in the text, including the Figure 3 legend, because as it stands, this is a misleading message.**

*Response: We are sorry about confusion over the term of “events averted” in our article. Yes, you are right. Everyone will die finally, either from CVD death or non-CVD death. We adopted this term from another study (*Bress AP, Kramer H, Khatib R, Beddhu S, Cheung AK, Hess R, Bansal VK, Cao G, Yee J, Moran AE, Durazo-Arvizu R, Muntner P, Cooper RS. Potential Deaths Averted and Serious Adverse Events Incurred From Adoption of the SPRINT (Systolic Blood Pressure Intervention Trial) Intensive Blood Pressure Regimen in the United States: Projections From NHANES (National Health and Nutrition Examination Survey). *Circulation.* 2017;135:1617-1628.*)*

*Thanks for your point. To avoid confusion, we would like to replace “event averted” with “events reduced” for the legend:*

*“The ’events reduced’ is calculated by multiplying the event difference from our simulation with the number of SPRINT eligible adults.”*

6. **Original comment**

**Comment on Table 1: It is still unclear what costs are presented in column named "Costs, Int $". Are these hospital costs, or do they include treatment costs? Regardless, it would be very helpful to look separately at hospital costs and treatment costs.**

**Revision 3 coment**

**Could the authors please incorporate their answers (ie explanation of what costs are presented) into the manuscript text, including the Table caption or legend. Secondly, while I appreciate that cost information on specific diagnosis codes may be difficult to obtain, splitting the costs by total treatment costs vs total event costs should be very easy to do from the model – indeed, that is how total costs are usually calculated. It would provide a necessary context for the reader to understand what the cost differences are driven by, which, in turn would help place results in a general context (ie what happens if treatment intervention price changes etc)**

*Response: Many thanks for your points and advice.*

*We have added the following text in main text on [page 10 line 237-238]*

*“Total costs included antihypertension treatment costs, cost of complications and SAE costs. Specifically, for costs of blood pressure medications …”*

*and the table legend on [page 27]*

*Total costs included antihypertension treatment costs, cost of complications and serious adverse event costs.”*

*Meanwhile, we performed an analysis* *to split the costs by antihypertension treatment costs, cost of complications and SAE costs and added the following table in the appendix.*

*S8 Table	Total costs of intensive BP control and standard BP treatment*

|  | *Intensive BP control ^a^* | *Standard BP treatment ^a^* | *Difference ^b^* |
| --- | --- | --- | --- |
| *Antihypertension treatment costs* | *8690* | *4960* | *4039(395,8744)* |
| *Cost of complications* | *1774* | *2280* | *-570(-751,181)* |
| *SAE costs* | *931* | *612* | *308(257,359)* |
| *Total costs* | *11395* | *7861* | *3777(-208,8286)* |

*^a^ To convert cost input to Chinese currency, the cost was multiplied by purchasing power parity (PPP) rate (in this case, 3.54). Total cost includes antihypertension treatment costs, cost of complications and serious adverse event costs.*

*^b^*  *Probabilistic analyses will run the model 1000 times with the use of randomly selected values for input measurements from predefined distributions. The uncertainty intervals (UIs) show the 2.5 to 97.5 percentiles for the incremental differences in costs.*
