# Supplementary material for: Applicability and cost-effectiveness of the Systolic Blood Pressure Intervention Trial (SPRINT) in the Chinese population: A cost-effectiveness modeling study
Source: PLoS Med. 2021 Mar 4;18(3):e1003515. doi: 10.1371/journal.pmed.1003515 (PMC7971845; doi:10.1371/journal.pmed.1003515)
Supplement: Attachment — Submitted filename: Point-by-point responses PMEDICINE-D-20-01125R4_22Dec 2020.docx [file pmed.1003515.s011.docx]

**Requests from Editors:**

**1- Thank you for clarifying the data availability. Please also update the data availability statement in the submission metadata to read:
“CHARLS data are available via the website:** [**http://charls.pku.edu.cn/pages/data/2011-charls-wave1/en.html**](https://protect-eu.mimecast.com/s/IT4EC00N7SG8nWGUoBBv3?domain=charls.pku.edu.cn) **Other relevant data are within the manuscript and its Supporting Information files”**

*Response: Thank you for this suggestion. we have changed the statement as suggested on [page 7 line 168-169]:*

*“CHARLS data are available via the website:* [**http://charls.pku.edu.cn/pages/data/2011-charls-wave1/en.html**](https://protect-eu.mimecast.com/s/PwMaCOPjDUp4Wn7hpAvyk?domain=charls.pku.edu.cn)*. Other relevant data are within the manuscript and its Supporting Information files.”*

**2- In your response to Reviewer 3, comment 5, you change “events averted” to “events reduced”, but I’m not sure that clarifies what the figure is reporting. Please add a bit more detail to the description in the figure legend, so it’s clear what outcomes are represented in the figure.**

*Response: Many thanks for you points. we have added some words to clarify it.*

*Please find it below and also in the main text on [page 22 line 554-555]:*

*“The ’events reduced’ refers to events that would be reduced if the intensive treatment were in place instead of standard treatment. It is calculated by multiplying the event difference from our simulation with the number of SPRINT eligible adults.”*
